# FAN1 removes triplet repeat extrusions via a PCNA- and RFC-dependent mechanism

**DOI:** 10.1073/pnas.2302103120

**Published:** 2023-08-07

**Authors:** Ashutosh S. Phadte, Mayuri Bhatia, Hope Ebert, Haaris Abdullah, Essam Abed Elrazaq, Konstantin E. Komolov, Anna Pluciennik

**Affiliations:** ^a^Department of Biochemistry and Molecular Biology, Thomas Jefferson University, Philadelphia, PA 19107

**Keywords:** FAN1 nuclease, Huntington’s disease, DNA mismatch repair, MutS beta, triplet repeats

## Abstract

Human genome-wide association studies have identified *FAN1* and several DNA mismatch repair (MMR) genes as modifiers of Huntington’s disease age of onset. In animal models, FAN1 prevents somatic expansion of CAG triplet repeats, whereas MMR proteins promote this process. To understand the molecular basis of these opposing effects, we evaluated FAN1 nuclease function on DNA extrahelical extrusions that represent key intermediates in triplet repeat expansion. Here, we describe a strand-directed, extrusion-provoked nuclease function of FAN1 that is activated by RFC, PCNA, and ATP at physiological ionic strength. Activation of FAN1 in this manner results in DNA cleavage in the vicinity of triplet repeat extrahelical extrusions thereby leading to their removal in human cell extracts. The role of PCNA and RFC is to confer strand directionality to the FAN1 nuclease, and this reaction requires a physical interaction between PCNA and FAN1. Using cell extracts, we show that FAN1-dependent CAG extrusion removal relies on a very short patch excision-repair mechanism that competes with MutSβ-dependent MMR which is characterized by longer excision tracts. These results provide a mechanistic basis for the role of FAN1 in preventing repeat expansion and could explain the antagonistic effects of MMR and FAN1 in disease onset/progression.

## Significance

FAN1 is a DNA repair enzyme, and variants of FAN1 modify Huntington’s disease (HD) onset and progression. However, the biological function of FAN1 in HD etiology is poorly understood. Here, we show that FAN1 is a strand-directed nuclease that requires PCNA and RFC for its activation on DNAs harboring triplet repeat extrusions. This process competes with MutSβ-initiated DNA mismatch repair (MMR) both in minimal systems composed of purified proteins as well as in cell extracts and likely counteracts the causative role of the MMR system in repeat expansion. We propose that the balance between these opposing pathways likely determines the rate of repeat expansion and may be critical for the maintenance of genomic stability.

Repetitive DNA sequences constitute about half of the human genome and play important roles in the regulation of gene expression (1). Such sequences are genetically unstable, with higher instability associated with increased length and sequence homogeneity (2). Mutation rates within DNA repeats tend to be 10^1^ to 10^5^ fold higher than in other parts of the genome (1). Instabilities of tri-, tetra-, penta-, and hexa-nucleotide repeats in distinct and unrelated genes are associated with a number of neurodegenerative, musculoskeletal, and neurodevelopmental disorders (3). Although the molecular mechanisms of repeat instability are not completely understood, there is general agreement that the formation of transient DNA structures such as hairpin loops and extrahelical extrusions by strand mishybridization underlies repeat instability (4, 5).

Maintenance of genomic stability has necessitated the evolution of DNA repair mechanisms which act by rectifying DNA damage caused by endogenous and exogenous agents. DNA mismatch repair (MMR) is a highly conserved antimutagenic pathway that corrects replication errors and prevents chromosomal rearrangements (6, 7). Unexpectedly, a mutagenic noncanonical function of MMR has been implicated as the cause of triplet repeat expansions (8). Inactivation of DNA mismatch repair (MMR) genes (*MSH3*, *MSH2*, *MLH1*, *PMS2*, and *MLH3*) in cellular and animal models of neurological conditions such as Huntington’s disease (HD), myotonic dystrophy type1 (DM1), and fragile-X related disorders (FXDs) has been shown to attenuate triplet repeat expansion (reviewed in ref. 9).

Evidence for a role for MMR in neurodegenerative disease in humans has emerged from genome-wide association studies (GWAS) in HD patients wherein *MSH3*, *PMS1*, *PMS2,* and *MLH1* have been identified as modifiers of disease onset age (10–16). In addition to the MMR genes, *FAN1* (FANCD2 and FANCI-associated nuclease 1) (17) is a modifier not only of the age of HD onset but also of CAG and CGG repeat expansion (10, 15, 18–20). However, in contrast to MMR, knockout of *FAN1* exacerbates CAG and CGG repeat expansion (15, 19, 20), suggesting that the FAN1 and MMR pathways exert opposing effects, with FAN1 attenuating repeat expansion and MMR promoting it.

The best-studied function of FAN1 is in the removal of DNA interstrand crosslinks (ICLs) at stalled replication forks. The importance of FAN1 in this process is underscored by the observation that its inactivation results in increased sensitivity of human cells to the cytotoxic effects of ICL-inducing agents such as cisplatin and mitomycin C (17, 21, 22). The FAN1 enzyme possesses 5´flap endonuclease and 5´ to 3´ exonuclease activities (17, 21, 23, 24) suggesting roles in a range of DNA repair processes. In fact, recent studies have shown that FAN1 nuclease can also cleave long triplet repeat extrusions (25). While strategies that target these enzymatic activities could serve as therapeutic approaches for a variety of neurodegenerative diseases, the lack of a full mechanistic understanding of FAN1 function poses a significant impediment.

Here, we describe a unique FAN1-catalyzed (CAG)/(CTG) extrahelical extrusion cleavage activity that depends on the presence of FAN1, PCNA, RFC, and ATP and occurs at physiological ionic strength, suggesting a role for FAN1-containing multiprotein assemblies in modulation of triplet repeat expansion. This reaction occurs in a strand-directed manner such that cleavage occurs only when the extrusion and the strand break are located on the same DNA strand, raising the possibility that FAN1 may preferentially remove triplet repeat extrahelical extrusions from the nascent strand during DNA synthesis. We also show that FAN1 and MMR compete for occupancy of extrahelical extrusions, providing a molecular explanation for the opposing effects of MMR and FAN1 in repeat expansion and disease onset/progression.

## Results

### PCNA and RFC Activate FAN1 Nuclease on DNA Substrates Harboring (CAG)_2_ Extrahelical Extrusions.

**Fig. 1.**
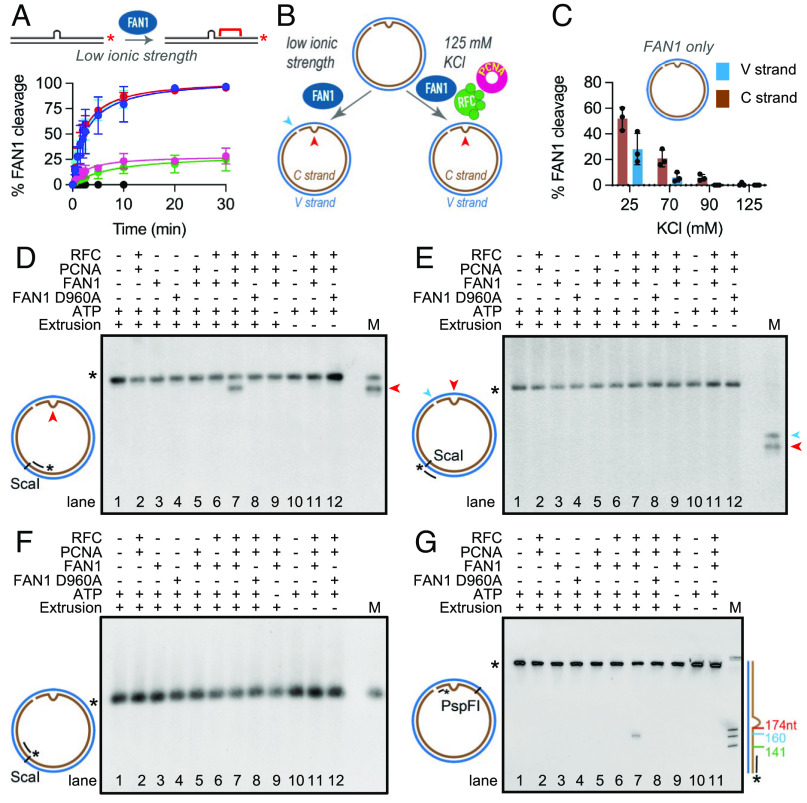
PCNA and RFC activate FAN1 nuclease on DNA substrates harboring (CAG)_2_ extrahelical extrusions. (*A*) Time course of FAN1 nuclease activity on 3′-/Cy3/-labeled 40-mer linear DNAs harboring different extrahelical extrusions [(CAG)_2_ (blue), (CTG)_2_ (cyan), (AGCCTA) (red), (CTG)_13_ (pink), (CAG)_13_ (green), and homoduplex control (black)] was performed at 70 mM KCl. Data are mean of at least three independent experiments. Error bars represent SD. (*B*) Schematic of the FAN1 nuclease assay on (CAG)_2_ extrusion harboring circular DNA under low or near-physiological ionic strength. The red and blue arrows indicate the location of the extrusion and the nick, respectively. (*C*) FAN1 cleavage of the C strand (brown) or the V strand (blue) of a 3′(CAG)_2_ DNA substrate was determined at different ionic strengths. Reaction products were digested with ScaI, resolved on 1% alkaline agarose gels, followed by indirect end labeling with 5′ digoxigenin (/5DigN/) labeled probe (Fwd1947) to visualize C strand (brown) or Rev1975 probe to visualize V strand (blue) (*SI Appendix*, *Materials and Methods* and Fig. S1*B*). Values are mean of n ≥ 3 independent experiments (± SD). (*D*) 3′(CAG)_2_ (lanes 1 to 9) or 3′ control homoduplex (lanes 10 to 12) were incubated in the presence or absence of FAN1 (or FAN1 D960A), PCNA, and RFC, as indicated. The reactions were performed at 125 mM KCl in the presence of ATP (except lane 9). Products were digested with ScaI, resolved on 1% alkaline agarose gels, followed by indirect end labeling with Fwd1947 probe, Rev1975 probe (*E*), or Fwd2020 probe (*F*). M- marker; mr78 4xLacO plasmid was digested with BglII (to indicate the location of (CAG)_2_ extrusion- red arrowhead) or BbvCI (to indicate the location of the nick- blue arrowhead). (*G*) Products of the reaction were also digested with PspFI and resolved on 10% polyacrylamide gels containing 8 M urea, followed by indirect end labeling with Fwd3028 probe. The size marker was generated by digestion of mr77 4xLacO with AlwNI, XbaI, or AatII with the distance from the nick indicated on the side. The mobility of the full-length-labeled DNA segment is indicated by asterisk. See also *SI Appendix*, Fig. S1.

To evaluate the role of FAN1 in the processing of CAG extrusions, we determined the substrate preference of FAN1 on linear heteroduplex DNA substrates that harbored various extrahelical extrusions. As shown in Fig. 1*A*, FAN1 cleaves (CAG)_2_ or (CTG)_2_ extrusions more efficiently than (CAG)_13_ and (CTG)_13_ loop-outs. To determine whether DNA sequence composition plays a role in activating FAN1 nuclease, we used a heteroduplex DNA substrate harboring a six-nucleotide extrusion composed of random sequence (AGCCTA). The efficiency of cleavage of this substrate by FAN1 was indistinguishable from that of the (CAG)_2_ and (CTG)_2_ extrusions. These observations suggest that FAN1 may recognize a wide range of extrahelical extrusions without regard to sequence composition. Since long CAG repeat tracts have a high propensity to form slipped-strand structures composed of small extrahelical extrusions, and because such extrusions are preferentially processed by MutSβ-dependent DNA mismatch repair (relative to the MutSα-dependent pathway), we decided to focus on circular DNA substrates harboring (CAG)_2_ or (CTG)_2_ extrusions. Therefore, we constructed circular double-stranded DNA substrates (the top strand of which is defined as the viral V-strand, and the bottom strand as the complementary C-strand) that harbor (i) a (CAG)_2_ extrahelical extrusion (on the C strand) and (ii) a defined single-strand break located 3′ to the extrusion also on the C strand. This substrate (designated as 3′(CAG)_2_) was incubated with recombinant full-length human FAN1 (*SI Appendix*, Fig. S1*A*) as depicted in the schematic in Fig. 1*B*. Products of the reaction were resolved on denaturing gels after ScaI cleavage, followed by Southern blot analysis (indirect end-labeling) using 5’-digoxigenin-labeled oligonucleotides that hybridize with either the C or V strand of the DNA substrate. At 25 mM KCl, we observed robust incision on the C-strand in the vicinity of the extrusion (as evidenced by a single cleavage product) (*SI Appendix*, Fig. S1 *B*, *Upper* lane 3 and Fig. 1*C*), as well as on the V-strand opposite to the nick (*SI Appendix*, Fig. S1 *B*, *Lower* lane 3 and Fig. 1*C*). FAN1 also cleaved 3’ homoduplex control DNAs, with cleavage, in this case, occurring primarily on the V-strand (opposite to the nick) (*SI Appendix*, Fig. S1 *B*, *Lower* lane 8, and *SI Appendix*, Fig. S1*C*). Similarly, we detected efficient FAN1 cleavage on both the C- and V-strands of the 5′(CAG)_2_ substrate (that harbored the extrusion on the C-strand and the nick on the V-strand) (*SI Appendix*, Fig. S1*D*), with limited cleavage observed on the 5′ homoduplex control (*SI Appendix*, Fig. S1*D*). FAN1 activity at 25 mM KCl was also observed on relaxed closed circular DNA substrates harboring (CAG)_2_ extrusion (and to a lesser extent on homoduplex control) (*SI Appendix*, Fig. S1 *E* and *F*). To further dissect the nature of FAN1 activity on such molecules, we analyzed the nuclease reaction products (at 70 mM KCl, where we observed significant nuclease activity on the (CAG)_2_ substrate, but very limited cleavage of the homoduplex control) by indirect end-labeling (*SI Appendix*, Fig. S1 *G* and *H*). Under these conditions, FAN1 nuclease was highly specific to the extrusion-containing strand, with the cleavage occurring in the vicinity of the extrusion. Our observations strongly suggest that FAN1 possesses an intrinsic strand preference that is guided by the presence of the extrusion. The incision activity observed on these molecules could be attributed to the FAN1 nuclease since the cleavage products were not evident when the wild-type enzyme was substituted by a nuclease-inactive variant of FAN1 (D960A) (21, 24) purified in a similar manner (*SI Appendix*, Fig. S1 *B* and *E*, lane 2). Interestingly, we observed that FAN1 nuclease activity was highly sensitive to ionic strength, with the efficiency of the reaction substantially diminished as a function of increasing monovalent salt concentration (*SI Appendix*, Fig. S1 *B* and *E*, lanes 3 to 6 and Fig. 1*C*). At 125 mM KCl in the presence of 5 mM MgCl_2_, FAN1 nuclease activity was below detection limits (*SI Appendix*, Fig. S1 *B* and *E*, lane 6), suggesting the requirement for additional cofactors to facilitate FAN1 activity under physiological ionic strength conditions (26).

The role of DNA-loaded PCNA (proliferating cell nuclear antigen) in activating latent nuclease functions of DNA repair proteins at physiological ionic strength has been documented previously (27–30). As exemplified by the case of MutLα (MLH1/PMS2 heterodimer), a physical interaction between the PCNA sliding clamp and the PMS2 subunit of MutLα is required for the activation of its endonuclease activity on mispair-containing substrates in a MutSα-dependent manner (31). Because the association between ubiquitinated PCNA and FAN1 has been reported previously (32), and since we observed extremely low levels of FAN1 nuclease activity at physiological ionic strength on both circular (Fig. 1*C* and *SI Appendix*, Fig. S1 *B*–*F*) and linear (see Fig. 3 *A*–*C*) extrusion-containing substrates, we evaluated the effects of PCNA on FAN1 nuclease activity. Addition of the clamp loader complex RFC (replication factor C) to the reaction facilitates the ATP-dependent loading of PCNA onto the circular, nick-containing DNA substrates (33). As shown in Fig. 1*D* (lane 7), incubation of a 3′(CAG)_2_ substrate with FAN1, PCNA, and RFC in the presence of ATP and Mg^2+^ at 125 mM KCl resulted in robust FAN1-dependent cleavage that is restricted to the strand harboring the CAG extrusion (compare lane 7 in Fig. 1 *D* and *E*). This process requires the catalytic activity of FAN1 since no cleavage was observed with the FAN1 D960A mutant (Fig. 1*D*, lane 8). Neither PCNA nor RFC were individually capable of activating FAN1 under these conditions (Fig. 1*D*, lanes 5, 6). Furthermore, omission of ATP from the reaction attenuated PCNA- and RFC-dependent FAN1 activation (Fig. 1*D*, lane 9). Because FAN1 activation in this manner requires both RFC, PCNA, as well as ATP, and since PCNA loading onto DNA by RFC occurs in an ATP-dependent manner (33), our findings support the idea that DNA-loaded PCNA is required for activation of FAN1 under physiological ionic strength conditions. It is noteworthy in this regard that ATP is not required for the intrinsic FAN1 catalytic activity (*SI Appendix*, Fig. S4*A*). Under these ionic strength conditions, distinct cleavage sites are observed proximal to the extrusion (compare Fig. 1 *D* and *F*), and no cleavage occurs on control homoduplexes (Fig. 1 *D*–*G*, lane 11). Polyacrylamide gel analyses of the FAN1 nuclease products revealed that the cleavage occurs between the strand break and the extrusion at a site that is ~14 nucleotides from the (CAG)_2_ (Fig. 1*G*).

### Strand Directionality of PCNA-, and RFC-Dependent Activation of the FAN1 Nuclease.

**Fig. 2.**
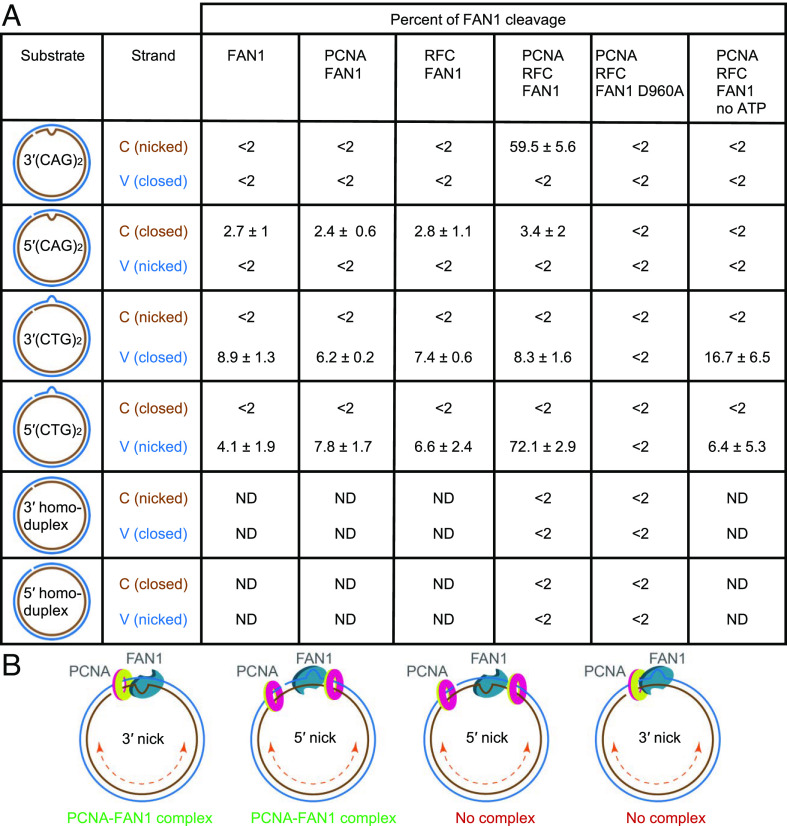
Strand directionality of PCNA- and RFC-dependent activation of the FAN1 nuclease. (*A*) FAN1 nuclease cleavage of C strand (brown) or V strand (blue) of extrusion-containing circular DNA substrates or homoduplex controls (as shown) in the presence of the indicated proteins was determined using indirect end labeling with Fwd1947 or Rev1975 probes, respectively, and quantified as described (*SI Appendix*, *Materials and Methods*). Data are mean of at least three independent experiments ± SD, except for 3′(CTG)_2_ DNA, presented data is an average of two independent experiments with range observed. Representative images are shown in Fig. 1 and *SI Appendix*, Fig. S2. ND, not determined. (*B*) Proposed mechanism for FAN1 interaction with DNA-loaded PCNA. On the 3′(CAG)_2_ or 5′(CTG)_2_ DNA substrates (strand break and the extrusion are on the same DNA strand), PCNA and FAN1 can form a complex leading to activation of FAN1 nuclease. On the 3′(CTG)_2_ or 5′(CAG)_2_ substrates (strand break and the extrusion are on the opposite DNA strands), PCNA and FAN1 complex does not form, and no DNA cleavage by FAN1 is observed. See also *SI Appendix*, Fig. S2.

**Fig. 3.**
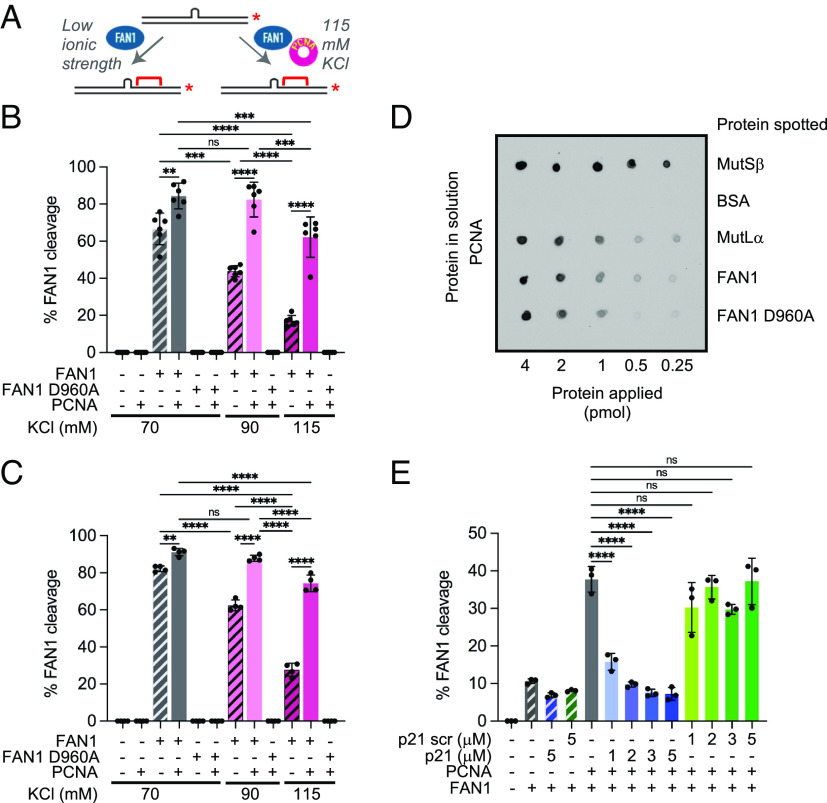
FAN1 nuclease activity is promoted by PCNA on linear DNA substrates harboring extrahelical extrusions. (*A*) Schematic of FAN1 activity in the presence or absence of PCNA on a 3′-/Cy3/-labeled 40 mer dsDNA harboring a (CAG)_2_ extrusion. Red brackets indicate approximate cleavage site(s). (*B*) Percentage of FAN1 cleavage on 3′-/Cy3/-labeled 40 mer harboring (CAG)_2_ extrusion in the presence or absence of PCNA at indicated KCl concentrations (see also *SI Appendix*, Fig. S3*A*). Experiment was repeated six times. **P < 0.01, ***P < 0.001, ****P < 0.0001, one-way ANOVA with post hoc Tukey’s test. Error bars represent SD. (*C*) Percentage of FAN1 cleavage on 3′-/Cy3/-labeled 40mer harboring (CTG)_2_ extrusion in the presence or absence of PCNA at indicated KCl concentrations. Experiment was repeated four times. **P < 0.01, ****P < 0.0001, ns- *P* > 0.05, one-way ANOVA with post hoc Tukey’s test. Error bars represent SD. (*D*) FAN1 physically interacts with PCNA. Indicated proteins were spotted onto nitrocellulose membrane and incubated with 0.36 µM PCNA overnight. PCNA was detected immunochemically (*SI Appendix*, *Materials and Methods*). (*E*) Quantification of percentage of FAN1 cleavage in the presence of PCNA and either p21 peptide or p21 scrambled peptide. Graph based on three independent experiments (±SD). ****P < 0.0001, ns- *P* > 0.05, one-way ANOVA with post hoc Dunnett’s test (comparison to FAN1 activity in the presence of PCNA only). See also *SI Appendix*, Fig. S3.

Our data demonstrate a new PCNA- and RFC-dependent activation of the FAN1 nuclease on 3′(CAG)_2_ substrates wherein the strand break and the extrusion are located on the same DNA strand (Fig. 1*D*, C strand). To determine whether the location of the strand break plays a role in controlling PCNA/RFC-dependent FAN1 nuclease activity, we prepared a 5′(CAG)_2_ substrate (Fig. 2, schematic) on which the nick and the extrusion are located on opposite strands. On this substrate, PCNA and RFC failed to activate FAN1 nuclease activity on either DNA strand (Fig. 2 and *SI Appendix*, Fig. S2 *A*–*C*). We reasoned that the lack of FAN1 activity on the 5′(CAG)_2_ substrate was either due to the polarity of the nick relative to the extrusion (3′ vs. 5′) or because of the placement of the nick and the extrusion on two different DNA strands (C-strand vs V-strand). To distinguish between these two scenarios, we evaluated FAN1 activity on a 5′(CTG)_2_ substrate wherein both the nick and the extrusion are on the same DNA strand (V strand) (Fig. 2, schematic). On this substrate, although low levels of FAN1 activity were detected (*SI Appendix*, Fig. S2 *D* and *E*, lanes 3, 5, 6, 9 and Fig. 2), a robust PCNA- and RFC-mediated stimulation of FAN1 cleavage was observed and this activity was restricted to the strand that contained both the extrusion and the nick (*SI Appendix*, Fig. S2 *D*–*G*, lane 7 and Fig. 2). By contrast, 3′(CTG)_2_ substrates (on which the extrusion is on the V strand and the nick is on the C strand) were refractory to PCNA and RFC stimulation of FAN1 (Fig. 2 and *SI Appendix*, Fig. S2 *H*–*J*). FAN1 nuclease displays a strict requirement for the presence of an extrusion since no FAN1 activity is detected on either 3′ or 5′ homoduplex controls (Fig. 2 and *SI Appendix*, Fig. S2), and it can efficiently cleave both (CTG)_2_ and (CAG)_2_ extrusion-containing DNA substrates (also refer to Fig. 3 *A*–*C*).

These data demonstrate for the first time that strand-specific PCNA/RFC-dependent FAN1 nuclease activity requires the strand break and the extrusion to be present on the same DNA strand regardless of whether the strand break is located 3′ or 5′ to the extrusion. Thus, the strand directionality of the reaction at physiological ionic strength is governed not only by the structure recognition properties of FAN1 nuclease itself but also by the strand break, PCNA, and RFC (Fig. 2*B*). Others have shown that strand breaks serve as sites for the loading of PCNA onto DNA by RFC (33, 34). Therefore, the simplest interpretation of our findings is that DNA-loaded PCNA activates the FAN1 nuclease via a physical interaction between these two proteins.

### FAN1 Nuclease Activity Is Promoted by PCNA on Linear DNA Substrates Harboring Extrahelical Extrusions.

To further dissect the roles of PCNA and RFC in FAN1 nuclease activation, we employed linear DNA substrates harboring (CAG)_2_ or (CTG)_2_ extrusions (Fig. 3*A*). The rationale for the use of linear DNAs comes from prior work demonstrating RFC-independent loading of PCNA via DNA ends (35). Indeed, we and others have shown that PCNA loaded onto DNA in this manner is sufficient to activate the MutLα endonuclease or DNA polymerase δ on linear DNA (29, 36). The FAN1 nuclease (but not FAN1 D960A) cleaved linear 40-bp DNA substrates harboring a (CAG)_2_ or (CTG)_2_ extrusions as judged by the appearance of a ~10-nucleotide hydrolytic product (*SI Appendix*, Fig. S3*A* and Fig. 3 *B* and *C*). The complementary DNA strand was not visibly cleaved (*SI Appendix*, Fig. S3*B*). As seen on circular DNAs (Fig. 1*C*), the FAN1 nuclease was sensitive to ionic strength, with >threefold reduction in its activity on extrusion-containing linear DNA substrates upon increasing monovalent salt concentration from 70 mM to 115 mM. Supplementation of the FAN1 nuclease reactions with PCNA (*SI Appendix*, Fig. S3*A*, compare lanes 3, 7, 10 to 4, 8, 11 and Fig. 3 *B* and *C*) resulted in rescue of the salt-dependent attenuation of the hydrolytic reaction. It is noteworthy that PCNA alone is sufficient to stimulate FAN1 activity on (CAG)_2_ extrusions and the presence of RFC does not further enhance the reaction (*SI Appendix*, Fig. S3*C*). Thus, RFC plays no significant role in FAN1 activation by PCNA in this system, and its role is likely limited to the loading of PCNA onto DNA. These findings also suggest that a physical interaction between FAN1 and PCNA may be required to stimulate FAN1 nuclease activity.

We have used two orthogonal methods to evaluate the FAN1–PCNA interaction. First, using far-western analysis, we observed a direct physical interaction between PCNA and FAN1 or FAN1 D960A (Fig. 3*D*) under conditions in which PCNA–MutSβ and PCNA–MutLα were also detected in line with previous observations (37). Second, surface plasmon resonance spectroscopy (SPRS) revealed that FAN1 interacts with sensor chip-bound PCNA (*SI Appendix*, Fig. S3 *D*, *Upper*), and that the interaction can be inhibited by a peptide harboring the PCNA-interacting motif (PIP-box) from the p21 protein (37–39). Disruption of the FAN1–PCNA interaction by the p21 peptide (but not a scrambled sequence control peptide) blocked PCNA-dependent activation of the FAN1 nuclease (Fig. 3*E* and *SI Appendix*, Fig. S3*E*). These results suggest that the activation of FAN1 by PCNA is mediated by a physical interaction between the two proteins.

Previous studies have indicated an interaction between ubiquitinated PCNA and FAN1 via a noncanonical PIP box located near the N-terminus of the protein (27-SNS*I*ISC*F*-34) (32). Therefore, the two crucial FAN1 residues (I30 and F34) that were shown to mediate the interaction with Ub-PCNA (32) were mutated to alanine. However, as judged by SPRS, recombinant mutated FAN1 I30A/F34A retained the ability to interact with PCNA, and the interaction was attenuated by the p21 peptide (*SI Appendix*, Fig. S3 *D*, *Lower*, compare solid and dashed lines). Consistent with these observations, FAN1 I30A/F34A nuclease was activated by PCNA on the linear DNA substrate harboring (CAG)_2_ extrusion in our two protein enzymatic reactions (*SI Appendix*, Fig. S3*F*). These findings suggest the existence of other uncharacterized PCNA binding site(s) on FAN1 that likely mediate the PCNA–FAN1 interaction.

### MutSβ Inhibits FAN1 Nuclease Activation on 3′(CAG)_2_ Substrate.

**Fig. 4.**
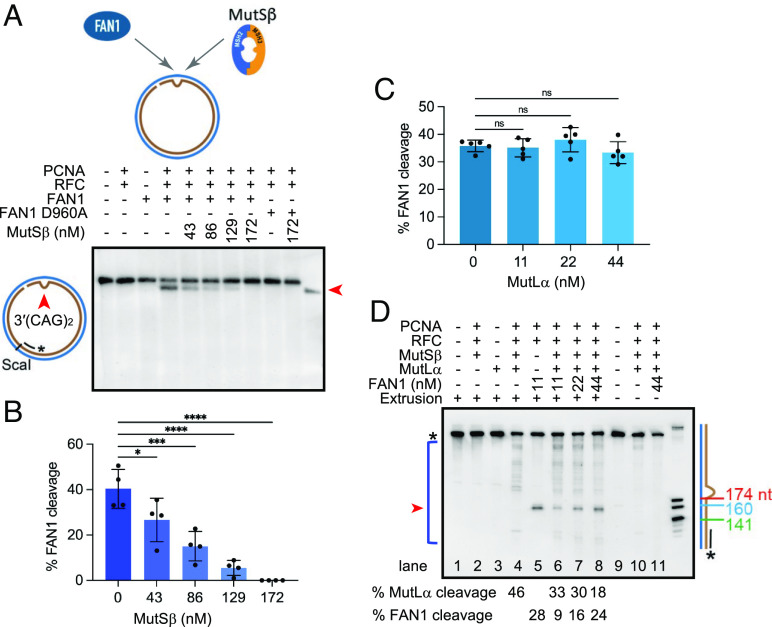
Effect of the components of DNA mismatch repair on FAN1 activity. (*A*) (CAG)_2_ extrusion harboring circular DNA substrate was incubated with FAN1 or (FAN1 D960A), PCNA, and RFC in the presence of increasing concentration of MutSβ (as indicated). Indirect end labeling was performed with Fwd1947 probe. (*B*) Percentage of FAN1 cleavage as in *A* based on four independent experiments with error bars representing SD. *P < 0.05, ***P < 0.001, ****P < 0.0001, 1-way ANOVA with post hoc Dunnett’s test (comparison to FAN1 in the absence of MutSβ). (*C*) Percentage of FAN1 cleavage of (CAG)_2_ extrusion-containing DNA substrate in the presence of PCNA and RFC and increasing concentration of MutLα (as indicated) and analyzed as in *A*. Quantification based on five independent experiments with error bars represent SD. ns- not significant, one-way ANOVA with post hoc Dunnett’s test (comparison to FAN1 in the absence of MutLα). (*D*) (CAG)_2_ extrusion DNA substrate was incubated with FAN1 or (FAN1 D960A), PCNA, RFC, MutSβ, and MutLα as indicated. Products of the reaction were digested with PspFI and resolved on 10% polyacrylamide gel containing 8 M urea. Indirect end labeling was performed with Fwd3028 probe. The size marker was generated by digestion of mr77 4xLacO with AlwNI, XbaI, or AatII. The distance of each DNA fragment from the nick is indicated on the side. The experiment was repeated twice with similar outcome. See also *SI Appendix*, Fig. S4.

Our previous studies demonstrated that MutSβ binds (CAG)_2_ extrusions with high affinity and specificity (28). Since (CAG)_2_ extrusions are also subject to recognition and cleavage by FAN1 nuclease, we asked whether occupancy of the (CAG)_2_ extrusion by MutSβ might attenuate PCNA-, RFC-, and ATP-dependent FAN1 nuclease activity on such DNAs. As shown in Fig. 4 *A* and *B*, increasing concentrations of MutSβ inhibited FAN1 nuclease activity on a 3′(CAG)_2_ DNA substrate, with near total inhibition observed upon addition of >130 nM MutSβ (~12-fold molar excess over FAN1). This result suggests that extrusion occupancy by MutSβ likely prevents FAN1 from processing such structures, although a role for a direct protein–protein interaction between MutSβ and FAN1 cannot be excluded. Furthermore, it could be that the PCNA–MutSβ and PCNA–FAN1 interactions modulate these effects. Therefore, we evaluated the effects of MutSβ on FAN1 nuclease at low ionic strength in the absence of PCNA. Preincubation of the (CAG)_2_ substrate with MutSβ resulted in ~50% inhibition of FAN1 nuclease activity (*SI Appendix*, Fig. S4*A*). We then took advantage of the known property of MutSβ to dissociate from an extrusion upon challenge with ATP (*SI Appendix*, Fig. S4*B*) (37, 40) to determine whether extrusion occupancy by MutSβ was responsible for the inhibition of FAN1. Indeed, the addition of 1 mM ATP resulted in complete restoration of FAN1 activity (*SI Appendix*, Fig. S4*A*). Consistent with these findings, (CAG)_2_ extrusion-bound MutSβ precludes binding of FAN1 (*SI Appendix*, Fig. S4*C*, lanes 3, 4), further suggesting that binding of MutSβ and FAN1 to the extrusion likely occurs in an either/or fashion. Unlike with MutSβ, preincubation of such DNAs with MutSα did not inhibit FAN1 nuclease activity (*SI Appendix*, Fig. S4*D*). These data suggest that FAN1 and MutSβ compete for occupancy of the CAG extrusion and raise the prospect that factors modulating this competition may drive CAG repeat expansion.

### MutLα Has Limited Effect on FAN1 Nuclease Activation on 3′(CAG)_2_ Substrate.

FAN1 was originally discovered as an interactor of MutL homologs MLH1 and PMS2 (components of MutLα) (41), and the motif on FAN1 that governs this interaction has been identified (42–44). In agreement with these studies, we observed an interaction between FAN1 and MutLα using far-western analysis (*SI Appendix*, Fig. S4*E*). Since it has been suggested that FAN1–MLH1 interaction may modulate processing of CAG repeats (42), we evaluated the effect of MutLα on FAN1 nuclease function. At physiological ionic strength, increasing concentrations of MutLα (up to fourfold molar excess over FAN1) had no significant effect on PCNA-activated FAN1 on circular DNAs containing a (CAG)_2_ extrusion (Fig. 4*C* and *SI Appendix*, Fig. S4*F*). Because MutLα does not display DNA binding activity in the absence of MutSα or MutSβ at physiological ionic strength (27, 45), these results suggest that the MutLα–FAN1 interaction does not affect FAN1 activity. This result is also recapitulated under low ionic strength conditions, where MutLα alone or in the presence of MutSα does not inhibit FAN1 nuclease activity (*SI Appendix*, Fig. S4 *G* and *H*, respectively) and is in agreement with recent findings (43).

### Effect of FAN1 on MutSβ-, PCNA-, and RFC-Dependent MutLα Endonuclease Activation on 3′(CAG)_2_ Extrusion Substrate.

Although our data point to competition for DNA extrusions between MutSβ and FAN1, it should be noted that MutSβ initiates MMR in concert with several other proteins. The first steps of the reaction involve activation of the MutLα endonuclease in a MutSβ- and heteroduplex-dependent manner in the presence of RFC, PCNA, and ATP (28), a reaction that has been reconstituted from purified components. As shown in Fig. 4*D* and *SI Appendix*, Fig. S4*I* (lane 4), we observed robust and multiple MutLα-catalyzed incisions of the extrusion-containing DNA substrate in the presence of MutSβ, MutLα, RFC, and PCNA in a buffer containing 125 mM KCl, 5 mM MgCl_2_, and 1.5 mM ATP, in line with previous work (27, 28). By contrast, RFC- and PCNA-dependent FAN1 nuclease cleavage of the substrate under these conditions results in the appearance of a single band (Fig. 4*D* and *SI Appendix*, Fig. S4*I*, lane 5). Thus, our assay permits the concurrent monitoring of both FAN1 and MutLα nuclease activities. As evident in Fig. 4*D* (lanes 5 and 6), addition of MutSβ and MutLα to this reaction resulted in robust MutLα catalyzed incisions while strongly reducing FAN1 activity by ~threefold; this inhibition was reversed by supplementation with an excess of FAN1. Taken together with the results from Fig. 4*A*, these findings strongly suggest that FAN1 and MutSβ-dependent MMR pathway compete for DNA extrusions and that local concentrations of the competing proteins may dictate pathway choice. Robust MutLα endonuclease activity was also observed on a 5′(CAG)_2_ substrate under these experimental conditions (*SI Appendix*, Fig. S4*J*, lane 4), in agreement with published work (27, 28). However, unlike for the 3′(CAG)_2_, addition of FAN1 to this reaction did not inhibit MutLα endonuclease activity (*SI Appendix*, Fig. S4*J*, lanes 6–8). These data are in agreement with our observation that FAN1 nuclease cleaves DNA harboring extrahelical extrusions when the extrusion and the nick are on the same DNA strand (Fig. 2).

### DNA Substrates Containing (CAG)_2_ Extrahelical Extrusion Are Subject to FAN1-Dependent Extrusion Removal in Nuclear Extracts.

**Fig. 5.**
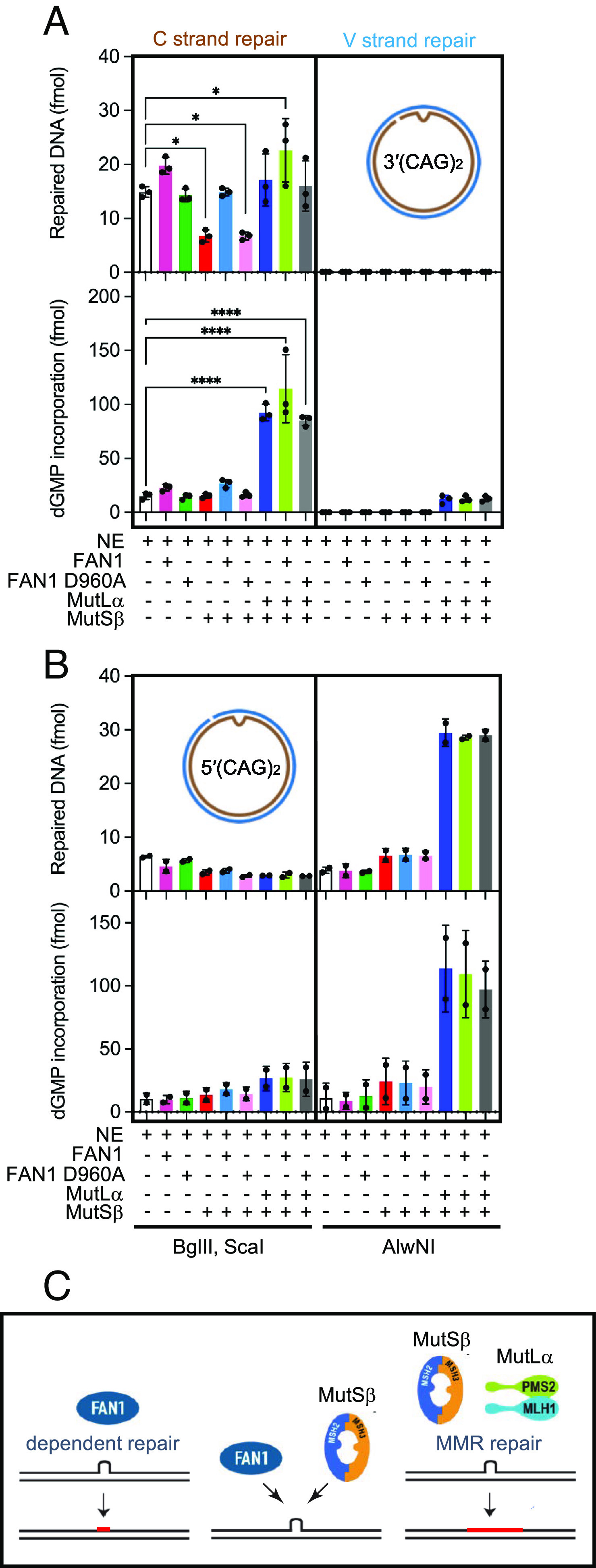
DNA substrates containing (CAG)_2_ extrahelical extrusion are subject to FAN1-dependent extrusion removal in nuclear extracts. (*A*) A 3′(CAG)_2_ DNA substrate was incubated in the presence of [α-^32^P] dGTP with nuclear extract derived from MLH1^−/−^ MSH3^−/−^ HCT116 cells, which was supplemented as indicated with FAN1, nuclease-dead FAN1 D960A, MutSβ, and MutLα. Repair was scored by cleavage with BglII and ScaI (C strand repair) or AlwNI (V strand repair). Repair products were visualized after staining with ethidium bromide (upper graph) while repair DNA synthesis was quantitated by exposure to phosphorimager screens (lower graph) (*SI Appendix*, *Materials and Methods*). Results are mean of three independent experiments (±SD). *P < 0.05, ****P < 0.0001, one-way ANOVA with post hoc Dunnett’s test (comparison to NE only). The [α-^32^P] dGMP incorporation indicated corresponds to that found in repair bands only (representative images are shown in *SI Appendix*, Fig. S5*A*). (*B*) A 5′(CAG)_2_ DNA substrate was incubated in the presence of [α-^32^P] dGTP with HCT116 nuclear extracts as in described in *A*. Repair levels of either DNA strand (upper graph) or repair DNA synthesis levels (lower graph) shown are average of two independent experiments with error bars representing ranges observed. Representative images are shown in *SI Appendix*, Fig. S5*F*. (*C*, *Left*) FAN1-dependent repair relies on short repair resynthesis tract (red line). (*C*, *Middle*) competition between MutSβ-dependent and FAN1-dependent repair pathways. (*C*, *Right*) MMR-dependent repair relies on long resynthesis tracts (red line). See also *SI Appendix*, Fig. S5.

To address the possibility that nick-dependent FAN1 nuclease cleavage of extrahelical extrusions might lead to removal of such structures, we evaluated the fate of 3′(CAG)_2_ or 5′(CAG)_2_ DNAs upon incubation with nuclear extracts of HCT116 tumor cells (MLH^-/-^ and MSH3^-/-^) that are deficient in MutLα and MutSβ (46). Removal of the (CAG)_2_ extrusion was monitored by cleavage with BglII or AlwNI endonucleases, and the extent of repair DNA synthesis was measured by incorporation of [α-^32^P] dGMP into newly synthesized DNA (*SI Appendix*, Fig. S5*A*, diagram). We observed efficient nick-directed removal of the extrusion from the 3′ (CAG)_2_ substrate (as judged by restoration of sensitivity to BglII restriction) even in the absence of MutLα and MutSβ (*SI Appendix*, Fig. S5*A*, upper image, lane 2 and Fig. 5*A* upper graph) as has been observed previously (28, 47, 48). This MMR-independent process relies on extrusion removal via short-patch repair, as evidenced by low levels of [α-^32^P] dGMP incorporation in the DNA repair products (*SI Appendix*, Fig. S5*A*, lower image, lane 2 and Fig. 5*A* lower graph). To establish whether this MMR-independent extrusion removal machinery includes FAN1, we carried out a DNA pulldown assay from HCT116 nuclear extracts and observed a threefold enrichment of FAN1 on 3′(CAG)_2_ substrate in comparison to homoduplex control DNA (*SI Appendix*, Fig. S5*B*, 10-min time point). Furthermore, partial immunodepletion of FAN1 from HCT116 nuclear extracts (*SI Appendix*, Fig. S5*C*) reduced the efficiency of MMR-independent extrusion removal by ~50% (in comparison to mock depletion experiment), an effect that was rescued by the addition of recombinant FAN1 to the reaction (*SI Appendix*, Fig. S5 *D* and *E*). These data suggest that the MMR-independent extrusion removal pathway depends at least in part on FAN1.

Interestingly, supplementation of HCT116 extracts with recombinant human MutSβ substantially (~50%) inhibited (CAG)_2_ extrusion removal, suggesting that MutSβ occupancy of the extrusion may interfere with MMR-independent repair [*SI Appendix*, Fig. S5*A*, lane 5, and Fig. 5*A* and (28)]. These observations are in line with the inhibitory effects of MutSβ on FAN1 nuclease activity shown in Fig. 4 *A* and *B* and *SI Appendix*, Fig. S4*A*. Further addition of FAN1 (but not FAN1 D960A) to MutSβ-supplemented HCT116 extracts counteracted the inhibitory effect of MutSβ on extrusion removal (*SI Appendix*, Fig. S5*A*, lanes 5, 6, 7, and Fig. 5*A*). Taken together, our findings are consistent with the idea that the MMR-independent CAG extrusion removal activity is at least in part catalyzed by FAN1 and that this repair process occurs without extensive excision and resynthesis.

Restoration of full MMR function to HCT116 nuclear extracts by supplementation with recombinant human MutSβ and MutLα (*SI Appendix*, Fig. S5*A*, lane 8 and Fig. 5*A* upper graph) resulted in robust extrusion removal that was accompanied by substantially higher repair DNA synthesis relative to extract alone (*SI Appendix*, Fig. S5 *A*, *Lower*, lanes 2 and 8, and Fig. 5*A* lower graph), consistent with the long excision and resynthesis tracts characteristic of MMR (49). It should be noted that repair (and associated repair DNA synthesis) of the 3′(CAG)_2_ substrate was biased to the nick-containing strand (*SI Appendix*, Fig. S5*A*, lanes 12 to 20).

As described in Fig. 2, CAG extrusion cleavage by FAN1 requires that the nick and the extrusion be contained on the same DNA strand. Thus, we hypothesized that if MMR-independent extrusion removal relies on FAN1 activity, the 5′(CAG)_2_ substrate would be refractory to repair in HCT116 extracts. Consistent with this prediction, we did not observe appreciable nick-directed repair of the 5′(CAG)_2_ substrate in these extracts (*SI Appendix*, Fig. S5*F*, lane 12 and Fig. 5*B* upper graph); however, robust repair and repair DNA synthesis was observed upon addition of MutSβ and MutLα to the reaction (*SI Appendix*, Fig. S5*D*, lane 18 and Fig. 5*B* lower graph), in agreement with previous findings (28). Thus, we propose that MMR-independent extrusion removal in HCT116 extracts is mediated by FAN1.

## Discussion

FAN1 was originally identified as a protein involved in the repair of DNA (ICLs) at stalled replication forks (17, 23, 50, 51), and loss of FAN1 function results in chromosomal instability and hypersensitivity of cells to ICL-inducing agents (17, 21, 22, 24, 52, 53). FAN1 deficiency in humans results in karyomegalic interstitial nephritis, a rare inherited kidney disorder that results in renal failure, a pathology recapitulated in *Fan1* knockout mice (52, 54–56). At the molecular level, FAN1 cleaves branched DNA structures with a preference for 5´flaps. It also possesses a 5´ to 3´ exonuclease activity on a variety of double- and single-stranded DNAs (17, 21, 23, 24).

More recently, GWAS have uncovered a role for FAN1 as a genetic modifier of HD onset, with loss of function variants hastening disease manifestation (10, 15, 18). In mouse models of HD, knockout of FAN1 promotes somatic CAG repeat expansion in the striatum, a brain region that is associated with disease pathophysiology in humans (19). In addition, FAN1 prevents CGG repeat expansions in a mouse model of FXDs (20, 57), suggesting a global role for this protein in the maintenance of genome stability. However, the molecular basis of FAN1 function in triplet repeat expansion and neurodegeneration remains unknown.

We have shown a new, PCNA-, and RFC-dependent, nick-directed FAN1 nuclease activity that is provoked by (CAG)_2_ or (CTG)_2_ extrahelical extrusions at physiological ionic strength. The FAN1 nuclease on its own is highly sensitive to monovalent salt concentration and, indeed, previous studies of full-length FAN1 employed low ionic strength conditions (25 to 70 mM NaCl or KCl) to detect nuclease activity (17, 21, 23, 25, 50, 58). We have found that under near physiological conditions (125 mM KCl and 5 mM MgCl_2_), FAN1 is activated on CAG or CTG extrahelical extrusions by PCNA and RFC. The role of RFC in this reaction is likely restricted to the loading of PCNA as evidenced by our findings on linear duplexes and by our demonstration that disruption of the physical interaction between PCNA and FAN1 is sufficient to abrogate the stimulation. It is noteworthy that nuclease activation at physiological ionic strength by DNA-loaded PCNA has been documented previously (27, 29, 30). The interaction of FAN1 with ubiquitinated PCNA has been documented in the context of stalled replication forks (32). Association with Ub-PCNA is mediated by a noncanonical PIP box adjacent to the UBZ domain of FAN1. Our findings suggest that FAN1 can also interact with nonubiquitinated PCNA via an as-yet unidentified PIP box, which we are currently mapping.

We demonstrate here that activation of FAN1 by PCNA occurs in a strand-directed manner, such that FAN1 cleavage occurs only when the nick and the extrahelical extrusion are on the same DNA strand (3′(CAG)_2_ and 5′(CTG)_2_ substrates) (Fig. 2). We attribute this effect to a combination of the asymmetric nature of PCNA loading onto the DNA, and the specific spatial orientation of FAN1 at the extrusion. The PCNA sliding clamp has two nonequivalent faces that are oriented uniquely relative to the 3′ double-strand–single-strand junction (38, 59–61) and Fig. 2*B*. Because the p21 peptide (which preferentially associates with one face of the sliding clamp (38)) blocks PCNA activation of FAN1 at near-physiological ionic strength, we conclude that FAN1 interacts with the same face of the clamp. Thus, the directionality of the loaded clamp confers an intrinsic asymmetry to the PCNA–FAN1 complex, which can form only when the nick and the extrusion are on the same DNA strand (Fig. 2*B*, two left panels). When the nick and the extrusion are on opposite strands (Fig. 2*B*, two right panels), the requirement for FAN1 to be in a unique orientation relative to the extrusion precludes the formation of the PCNA–FAN1 complex. Therefore, even though loaded PCNA can retain its spatial asymmetry by sliding along DNA (34) (Fig. 2*B*), it is unable to activate FAN1 on 5′(CAG)_2_ and 3′(CTG)_2_ DNA substrates. The role of the nick in this reaction is two-fold: to facilitate the loading of PCNA onto DNA and to confer orientation specificity to the loaded PCNA molecule. Thus, DNA-loaded, oriented PCNA may direct the FAN1 nuclease to extrusions that form by DNA polymerase slippage on the nascent strand during replication or repair.

The strand directionality mechanism discussed above is also recapitulated in experiments using nuclear extracts wherein CAG extrusions are removed by a FAN1-dependent process only when the extrusion and the nick are on the same DNA strand. In contrast, DNA mismatch repair is strictly restricted to the nick-containing strand regardless of which strand contains the extrusion (Fig. 5). Because PCNA can also be loaded onto DNA molecules harboring an extrahelical extrusion even in the absence of a nick, it is conceivable that PCNA loaded in this manner may activate FAN1 at DNA extrusions that form on resting DNA. Our observation that an extrusion composed of random sequence DNA is also susceptible to FAN1 cleavage suggests that PCNA-activated FAN1 may be involved in the removal of DNA extrusions of all types across the genome (including triplet repeat extrusions in post-mitotic neurons).

In this study, we have focused on DNA substrates harboring small extrahelical extrusions ((CAG)_2_ or (CTG)_2_). This choice was guided by observations in mouse models of HD and HD-patient-derived iPS cells and iPS-differentiated to medium spiny neurons, wherein expansion of the CAG repeat tract occurs in small size increments of approximately 2 CAG units per month (15, 62–64). We have previously shown that small CAG or CTG extrahelical extrusions provoke MutSβ-dependent initiation of DNA mismatch repair (activation of MutLα endonuclease) (28). Our findings that small CAG or CTG extrusions are also subject to cleavage by FAN1 raises the following question: Do FAN1 and MutSβ compete for occupancy of the extrusion? In support of this idea, we observe that FAN1 nuclease cleavage of extrahelical extrusions is subject to attenuation by MutSβ (but not MutSα). Furthermore, this competition also occurs in the context of MutSβ-dependent MutLα endonuclease activation and during the removal of the extrusion in nuclear extracts of human cells (Figs. 4 and 5). In fact, we find that cells lacking MutSβ rely on a FAN1-dependent extrusion removal process that exclusively employs a short-patch DNA excision and resynthesis mechanism that may involve other nuclease, DNA polymerase, and ligase activities. The reconstitution of a FAN1-dependent extrusion removal activity is an area of active interest for us. By contrast, in the presence of active DNA mismatch repair, removal of these extrusions occurs primarily via a long-patch DNA excision/resynthesis mechanism (Fig. 5) (27, 28, 49), although a short-patch mismatch repair reaction has also been described (65, 66). Interestingly, recent studies have postulated that modulation of CAG repeat expansion occurs by sequestration of MutLα by FAN1 via MLH1–FAN1 interaction (42, 43). Our data complements these studies and provides support for the view that competition between FAN1 and the MMR pathway may also be exercised through competition for extrusion occupancy. It is noteworthy that MLH1 effects on repeat expansion may also be mediated via MutLγ (MLH1/MLH3) as has been suggested by recent biochemical and genetic studies (67–71). We are experimentally pursuing this possibility.

Our findings described here may have broader implications for the mechanisms of triplet repeat expansion since they provide a molecular explanation for the opposing effects of MutSβ and FAN1 on somatic expansion of triplet repeats. Accordingly, CAG or CTG extrusion can be acted upon by either the FAN1-dependent pathway or MMR, thus maintaining a steady-state length of the CAG repeat tract. However, under conditions of MSH3 insufficiency, we postulate that CAG or CTG extrusions are removed by FAN1, thus exerting a “downward pressure” on repeat length. The short-patch nature of the repair DNA synthesis in this mechanism limits opportunities for strand slippage events that might lead to repeat expansion (Fig. 5*C*). This model is in agreement with the observation that knockout of MSH3 in mouse models of HD and DM1 prevents somatic expansions of the CAG or CTG tracts (62, 72).

The results described here provide the first mechanistic insight into the crosstalk between FAN1 and MMR pathways. Since CAG extrusions are subject to processing by either of these pathways, factors that modulate levels and/or activities of individual proteins might drive pathway choice and thereby repeat expansion.

## Materials and Methods

Detailed materials and methods are described in *SI Appendix*, *Materials and Methods*.

### DNA Substrates and Proteins.

Circular or linear heteroduplex DNA substrates and recombinant proteins used in this study were prepared as per previously established procedures with details provided in *SI Appendix*, *Materials and Methods*: *Proteins, cells and DNAs*.

### Nuclease Assays.

FAN1 nuclease assays on end-labeled linear DNA substrates were carried out by electrophoretic analyses of the products of DNA hydrolysis as described in **SI Appendix*, Materials and Methods: FAN1 nuclease assays on oligonucleotide DNA substrates.* Nuclease assays on circular DNA substrates were done by incubation with the indicated recombinant proteins, followed by indirect end-labeling of electrophoretically separated reaction products as detailed in **SI Appendix*, Materials and Methods: Nuclease assays on circular DNA substrates.*

### DNA Repair Assays.

Repair of extrahelical extrusions by HCT116 nuclear extracts was evaluated in the presence or absence of the indicated recombinant proteins as described in **SI Appendix*, Materials and Methods: DNA repair and repair synthesis in nuclear extracts.*

### Protein–Protein Interactions.

Interaction between FAN1 and PCNA was evaluated by far-western analysis and SPRS as described in *SI Appendix*, *Materials and Methods*: *Far western blot analysis and SPRS*, respectively.

## Supplementary Material

Appendix 01 (PDF)

## Data Availability

All study data are included in the article and/or *SI Appendix*.
