# Supplementary material for: FAN1 removes triplet repeat extrusions via a PCNA- and RFC-dependent mechanism
Source: Proc Natl Acad Sci U S A. 2023 Aug 7;120(33):e2302103120. doi: 10.1073/pnas.2302103120 (PMC10438374; doi:10.1073/pnas.2302103120)
Supplement: Supplementary file 1 — Appendix 01 (PDF) [file pnas.2302103120.sapp.pdf]

PNAS

Supporting Information for
FAN1 removes triplet repeat extrusions via a PCNA and RFC-dependent mechanism.

Ashutosh S. Phadte[1], Mayuri Bhatia[1], Hope Ebert[1], Haaris Abdullah[1], Essam Abed Elrazaq[1],

Konstantin E. Komolov[1] and Anna Pluciennik[1,*]

Anna Pluciennik
Email: anna.pluciennik@jefferson.edu

This PDF file includes:

Supporting text
Figures S1 to S5
Tables S1 to S3
SI References

Supporting Information Text

Materials and Methods

Proteins, cells and DNAs. Protein preparations used in this study are shown in **Fig. S1A**, and unless specified otherwise purity in each case exceeded 95%. Recombinant human MutLα and MutSβ, were purified from baculovirus infected Sf9 cells according to published protocols (1, 2). Human replication factor C (RFC) (3) was purified from baculovirus infected Sf9 cells using anti-FLAG M2 agarose, followed by Heparin HP and size exclusion chromatography on Superdex 200. Recombinant human proliferating cell nuclear antigen (PCNA) was prepared from *E. coli* harboring plasmid pET11a-PCNA and purified by published method (4). Recombinant human FAN1 was purified from *E. coli* cells harboring pET28a-His-MBP-FAN1 plasmid (synthesized by Genscript). FAN1 was purified through His-Trap HP column chromatography, followed by Heparin HP column chromatography, His-MBP tag removal, a second passing through His-Trap HP column, and size exclusion chromatography on Superdex 200. His-MBP tag removal resulted in FAN1 protein that has two additional amino acids SG in the N-terminal end of the protein and lacks the first Methionine (protein purity 93%). The nuclease dead mutant of FAN1, FAN1 D960A was generated by site directed mutagenesis. Briefly, mutagenesis was carried out using the Quikchange II site directed mutagenesis kit (Agilent, cat # 200517) using the primer pair 5'-GTGGCCTGCCGGCCCTGGTGGTTT-3' (forward) and 5'-AAACCACCAGGGCCGGCAGGCCAC-3' (reverse) in accordance with the manufacturer's instructions. The missense mutation results in the substitution of a key aspartate residue (D960) to alanine (GAC to GCC substitution) predicted to be responsible for enzyme catalysis (5, 6). The mutation was confirmed by sequencing of both DNA strands of the whole gene. The FAN1 I30A (ATC to GCC substitution) F34A (TTC to GCC substitution) DNA construct was generated at Genscript. Both proteins were purified using the same protocol as for wild type FAN1. All proteins were purified at Curia Global Inc. as a fee for service, while MutSα was purchased from Curia. HCT116 cells (purchased from ATCC, cat # CCL-247) were cultured and nuclear extracts prepared as described previously (7).

Circular single stranded DNAs (ssDNA) were isolated from E. coli SS320 cells (Lucigen, cat # 60512-1) carrying m13cp plasmid (8), while replicative form DNAs were isolated from E. coli XL1-blue cells. Heteroduplex and homoduplex controls were prepared by hybridization of ScaI-linearized replicative form and 4-6 fold excess of the appropriate circular ssDNA (**Table S1** and **Table S2**), followed by DNA ligation and isolation of relaxed closed circular (RCC) DNA by CsCl-ethidium bromide centrifugation as described previously (9). Nicked substrates, with the break 181 nt 5′ or 178 nt 3′ to the mismatch, were generated from RCC DNAs using Nt.BbvCI (New England Biolabs, cat # R0632S) or Nb.BbvCI (New England Biolabs, cat # R0631S), respectively.

Oligonucleotide based substrates harboring $(CAG)_2$, $(CTG)_2$ (AGCCTA), $(CAG)_{13}$, $(CTG)_{13}$ or homoduplex controls were prepared by annealing of HPLC-purified ssDNA complementary oligonucleotides (Integrated DNA Technologies) (**Table S3**). The annealing efficiency was assessed by electrophoresis on 10% polyacrylamide gels in TBE buffer. Annealed DNA substrates were aliquoted and stored at - 80 ºC until use.

Synthetic peptide (KRRQTSMTDFYHSKRRLIFS) containing the p21 C-terminal PCNA interaction element (10) and a scrambled sequence peptide (YDRSKLRTQSHRSFKTIMRF) were purchased from Genscript.

Far western blot analysis. Far western analyses were performed by spotting 0.25–4 pmol of the indicated proteins on a nitrocellulose membrane (Whatman). After incubation in blocking buffer (10 mM Tris-HCl, pH 8.0, 150 mM NaCl, 1 mM EDTA, 0.1% Triton X-100, and 5% milk solids) for 1 h at room temperature, the membrane was incubated overnight at 4 °C with 0.36 μM PCNA or 0.36 μM MutLα in blocking buffer as indicated, followed by three buffer washes. The presence of bound PCNA or MutLα was detected immunochemically with anti-PCNA (Santa Cruz Biotechnology, cat # sc-56) or anti-MLH1 (Abcam, cat # ab92312) antibodies.

FAN1 nuclease assays on oligonucleotide DNA substrates. Reactions (20 μL) contained 50 nM 3'-/Cy3/-labeled DNA homoduplex, $(CAG)_2$ or $(CTG)_2$, (AGCCTA), $(CAG)_{13}$, or $(CTG)_{13}$ extrusion harboring substrates, 250 nM FAN1 (or FAN1 D960A, or FAN1 I30A/F34A), 333 nM

PCNA (trimer) (or as indicated) in 25 mM HEPES-KOH pH 7.5, 5 mM $MgCl_2$, 0.05 mg/mL BSA (Sigma Aldrich, cat # 10711454001), and KCl as indicated. The reaction was preincubated in the absence of FAN1 for 10 min at 37 °C, followed by addition of FAN1 or its mutants (FAN1 D960A, FAN1 I30A/F34A) and further incubation for 5 min at 37 °C. Reactions were terminated using formamide (Fisher, cat # J67206.AE) at a final concentration of 70%. Samples were boiled at 95°C for five minutes and resolved by electrophoresis through 15% polyacrylamide gels containing 8 M urea (ThermoFisher, cat # EC68855BOX) in 45 mM Tris-borate, 1 mM EDTA, pH 8.0 (TBE buffer), and visualized using the BioRad ChemiDoc™ imaging system (BioRad, cat # 12003153). Percentage of FAN1 nuclease activity was interpreted as ratio of the intensity of the Cy3 signal in the cleavage products to the total signal intensity in the lane using ImageJ.

Effect of MutSβ or MutSα on FAN1 mediated cleavage. Reactions (20 μL) contained 50 nM 3'-/Cy3/-labeled DNA homoduplex or $(CAG)_2$. extrusion harboring substrates, 250 nM FAN1 (or FAN1 D960A), 125 nM MutSβ or MutSα, 1 mM ATP (ThermoFisher cat # R0441) as indicated, in the buffer containing 25 mM HEPES-KOH pH 7.5, 5 mM $MgCl_2$, 0.05 mg/mL BSA, 70 mM KCl. The reaction was preincubated for 10 minutes on ice, followed by incubation for 5 minutes at room temperature. FAN1 (or FAN1 D960A) was added and samples were incubated at 37 °C for 5 minutes and processed as described above.

Effect of MutLα in the presence or absence of MutSα on FAN1 activity. Reactions (20 μL) contained 50 nM of 3'-/Cy3/-labeled $(CAG)_2$ DNA substrate, 100 nM FAN1 (or FAN1 D960A), 100 nM MutSα, and MutLα as indicated, in 25 mM HEPES-KOH pH 7.5, 5 mM $MgCl_2$, 0.05 mg/mL BSA and 70 mM KCl. The reaction was preincubated with defined concentrations of MutLα for 10 min at 37 °C, followed by addition of FAN1 and further incubation for 5 min at 37 °C. The reactions were terminated and analyzed as described above.

Effect of RFC on PCNA-dependent FAN1 activity. Reactions (20 μL) contained 50 nM of 3'-/Cy3/-labeled $(CAG)_2$ DNA substrate, 250 nM FAN1 (or FAN1 D960A), 333 nM PCNA (trimer), 60 nM RFC and 1 mM ATP as indicated, in 25 mM HEPES-KOH pH 7.5, 5 mM $MgCl_2$, 0.05 mg/mL BSA and 115 mM KCl. Reaction mixes were preincubated with PCNA and/or RFC for 10 min at 37 °C, followed by addition of FAN1 and further incubation for 5 min at 37 °C. The reactions were terminated and analyzed as described above.

Effect of p21 or p21 scrambled peptides on PCNA-dependent FAN1 activity. Reactions (20 μL) contained 50 nM of 3'-/Cy3/-labeled $(CAG)_2$ DNA substrate, 100 nM FAN1 (or FAN1 D960A), 133 nM PCNA (trimer), indicated concentration of p21 or p21 scrambled peptide in 25 mM HEPES-KOH pH 7.5, 5 mM $MgCl_2$, 0.05 mg/mL BSA and 115 mM KCl. The reaction was preincubated in the absence of FAN1 for 5 min on ice, followed by addition of FAN1 and further incubation for 10 min at 37 °C. The reactions were terminated and analyzed as described above.

Nuclease assays on circular DNA substrates. Reactions (20 μL) contained 2.5 nM of 3'$(CAG)_2$, 5'$(CAG)_2$, 3'$(CTG)_2$, 5'$(CTG)_2$, 3' homoduplex or 5' homoduplex, 11 nM FAN1 (or FAN1 D960A) or as indicated, 36.5 nM PCNA (trimer), 10.5 nM RFC, 43 nM MutSβ, 15 nM MutLα and 1.5 mM ATP or as indicated, in 20 mM Tris-HCl pH 7.6, 1 mM glutathione (Millipore Sigma, cat # G4251), 0.05 mg/mL BSA, 2.5% glycerol, 5 mM $MgCl_2$, and 125 mM KCl. Reactions were incubated for 30 min at 37 °C and terminated by the addition of 2 μL of 1 mg/mL Proteinase K (Sigma Aldrich, cat # 3115887001), 140 mM EDTA, 1 % SDS, 1 mg/mL glycogen (Sigma Aldrich cat # 10901393001). Reactions were incubated at 56 °C for 30 minutes, followed by phenol: chloroform: isoamyl alcohol extraction and ethanol precipitation. For the salt dependence experiments, 2.5 nM of 3'$(CAG)_2$, 5'$(CAG)_2$, RCC $(CAG)_2$, 3' homoduplex, 5' homoduplex or RCC homoduplex were incubated at 37 °C for 30 min (or as indicated) in 20 μL reaction containing 11 nM FAN1 (or as indicated) in a buffer consisting of 25 mM HEPES-KOH pH 7.5, 1 mM glutathione, 0.05 mg/mL BSA, 4% glycerol, 5 mM $MgCl_2$, 1.5 mM ATP and KCl as indicated. Reactions were terminated as described above. Recovered DNA was linearized with ScaI (ThermoFisher, cat # FD0434). The digested products were resolved on 1 % alkaline agarose gels in 50 mM NaOH, 1 mM EDTA, transferred onto a Hybond N^+ membrane (Cytiva, cat # RPN203B), followed by Southern hybridization with 5'-digoxigenin labeled (/5DigN/) oligonucleotide probes (**Table S3**). Membranes were then incubated with anti-digoxigenin antibody (Millipore Sigma, cat # 11093274910) and the signal was detected using commercially

available reaction and detection kit (Millipore Sigma, cat # 11585762001) using BioRad ChemiDoc^{TM} imaging system. Alternatively, samples were linearized using a combination of ScaI and PspFI (ThermoFisher, cat # FD2224) and resolved on 10% polyacrylamide gels containing 8 M urea in TBE buffer, followed by electro-transfer onto a Hybond N^+ membrane, and analyzed by Southern hybridization as described above. Percentage of FAN1 nuclease activity was interpreted as ratio of the intensity of the signal in the cleavage products to the total signal intensity in the lane using ImageJ.

Surface Plasmon Resonance Spectroscopy. Interaction between PCNA and FAN1 (or FAN1 I30A/F34A) was monitored using Biacore 3000 (GE Healthcare). PCNA was immobilized by amine coupling on Carboxymethylated-dextran matrix of the CM5 chip (Cytiva: BR100012) which was activated by flowing 200 μL of freshly prepared EDC/NHS mix (200 mM/50 mM; Cytiva amine coupling kit, BR100050) at a flow rate of 20 μL/min. Injection of 10μg/mL PCNA diluted in 10 mM sodium acetate, pH 4.0 yielded immobilization of. 4000 response units (RU) for FAN1 and 2600 RU for FAN1 I30A/F34A). The remaining reactive carboxy groups were blocked by flowing 1 M ethanolamine hydrochloride, pH 8.5, at 25 μL/min for 7 minutes. Binding of FAN1 (or FAN1 I30A/F34A) was analyzed in buffer containing 20 mM Tris-Cl pH 7.5, 70 mM NaCl, 10 mM $CaCl_2$, 1 mM EDTA pH 8.0, 0.01% Surfactant P20, 1 mM DTT and 0.05 mg/mL BSA. Hundred μL of 50 nM FAN1 or FAN1 I30A/F34A (in the presence or absence of 15 μM p21 peptide) diluted in the running buffer, was injected at a flow rate of 20 μL/min with a contact time of 300 seconds and dissociation time of 120 seconds. The blank flow cell response was used as a reference to subtract the non-specific response.

Electrophoretic mobility shift assays (EMSA). MutSβ binding to the extrusion containing DNA was assessed by EMSA. Twenty µL reaction mixes contained 50 nM of 3'/Cy3/-labeled $(CAG)_2$ or homoduplex DNA in 25 mM HEPES-KOH pH 7.5, 0.05 mg/mL BSA, 5 mM $MgCl_2$, 70 mM KCl and 5 % glycerol. Samples were supplemented with 1 mM ATP and/or 125 nM MutSβ as indicated. All reactions were incubated on ice for 10 minutes followed by 5 minutes at room temperature and were run on 6 % TBE native polyacrylamide gels in 0.5 X Tris-Borate-EDTA buffer and was visualized by monitoring the Cy3 label intensity.
To investigate the interplay between FAN1 and MutSβ for substrate occupancy, resolved products from EMSA were further screened by immunoblotting. Twenty µL reaction mixes contained 50 nM 3'/Cy3/-labeled DNA substrate in 50 mM Tris pH 8.0, 0.05 mg/mL BSA, 10 mM $CaCl_2$, 70 mM KCl, 25 ng poly(dI/dC) (Sigma Aldrich Cat # P4929) and 5 % glycerol. Reactions were preincubated with 125 nM MutSβ on ice for 10 min. Following preincubation, 125 nM FAN1 was added, and reaction mixes were immediately run on 4 % TB native polyacrylamide gels in 0.5 X Tris-Borate buffer. Gels were visualized by monitoring the Cy3 label intensity. Following EMSA, substrate occupancy by either or both FAN1 and MutSβ was assessed by two channel multiplex fluorescence following transfer of the resolved products onto Immobilon PVDF membrane. The membrane was blocked in blocking buffer (1X Tris Buffered Saline supplemented with 0.01 % Tween 20 (Fisher cat # BP337-500) and 5 % milk solids) for 1 hour, followed by overnight incubation with anti-FAN1 (1:1000, Proteintech cat # 17600-1-AP) and anti-MSH3 (1:1000, BD Biosciences cat # 611390) antibodies at 4 °C. Signal intensity corresponding to MutSβ was visualized using the IRDye® 680RD goat anti-mouse IgG secondary antibody (LICOR Biotech Cat # 926-68070), and that corresponding to FAN1 was visualized using the IRDye® 800CW goat anti-rabbit IgG secondary antibody (LICOR Biotech Cat # 926-32211). Images were captured on the LICOR Odyssey XF imaging system.

DNA repair and repair synthesis in nuclear extracts. $(CAG)_2$ extrusion repair in nuclear extracts of HCT116 cells ($MLH1^{-/-}$, $MSH3^{-/-}$) was determined in 20 µL reactions containing 2.5 nM 3'$(CAG)_2$ or 5'$(CAG)_2$ (or homoduplex control) DNA substrates; 20 mM Tris-HCl pH 7.6; 110 mM KCl; 5 mM $MgCl_2$; 1.5 mM ATP; 1 mM glutathione; 0.05 mg/mL BSA; 0.1 mM each dATP, dGTP, dTTP, dCTP, and [α-^{32}P] dGTP (1 µCi/nmol); and 50 µg nuclear extract. Reactions were supplemented with 11 nM FAN1 (or FAN1 D960A), 43 nM MutSβ and 28 nM MutLα, as indicated. After incubation at 37 °C for 30 min, heteroduplex repair was scored by digestion with BglII and

ScaI or with AlwNI. Digestion products were separated on 1% agarose gels in 40 mM Tris-acetate, 1 mM EDTA at pH 8.2, and visualized after ethidium staining. Gels were then dried and exposed to phosphorimager screens along with [α-^{32}P] dGTP standards and quantitated using a Molecular Dynamics PhosphorImager. [^{32}P] dGMP incorporation was determined by comparison of the signal from repaired DNA to [α-^{32}P] dGTP standards.

DNA pulldown assays. 3'$(CAG)_2$ or 3' homoduplex control were immobilized to M-280 streptavidin beads (Invitrogen, cat # 11205D) with purified biotinylated Lac repressor protein (Bio-LacI) (protein purified as described in (11)). Such immobilized DNA substrates were then used for the DNA pulldown assays. Briefly, reactions (260 μL) contained 2.5 nM immobilized DNA substrate, 20 mM Tris-HCl, pH 7.6; 110 mM KCl; 5 mM $MgCl_2$; 1.5 mM ATP; 1 mM glutathione; 0.05 mg/mL BSA; 0.1 mM each dATP, dTTP, dCTP, dGTP; and 1300 μg HCT116 nuclear extract were incubated at 37 °C. Samples (60 μL) were withdrawn at indicated time points and placed on a magnet. The beads were then washed twice in a buffer composed of 20 mM Tris-HCl pH 7.6; 110 mM KCl; 5 mM $MgCl_2$; and 0.05 mg/mL BSA and resuspended in 20 μL of PBS, 1 mM DTT, 0.5% SDS. Samples were then heated at 65 °C for 5 min. Seven μL of the reactions were treated with proteinase K, followed by phenol:chloroform:isoamyl alcohol extraction and ethanol precipitation. Recovered DNA was resuspended in 10 μL of TE. Five μL of each DNA sample was linearized with ScaI, run on 1% agarose gel in the presence of 1μg/mL ethidium bromide to evaluate DNA recovery in each reaction (**Fig. S5B**, DNA panel). Five μL of recovered DNA from the 3'$(CAG)_2$ reactions was digested with BglII and ScaI to evaluate the level of repair (as described above) (**Fig. S5B**, DNA repair panel). Proteins from the remaining 13μL were eluted from dynabeads by boiling for 5 min at 95 °C in Laemmli buffer and analyzed by SDS-PAGE (along with 50 μg or 100μg of HCT116 nuclear extract as well as human recombinant FAN1 as controls), followed by Western blot using anti-FAN1 antibodies (Proteintech, cat # 17600-1-AP) or anti-PCNA antibodies (Santa Cruz Biotechnology, cat # sc-56) (as indicated in **Fig. S5B**).

FAN1 immunodepletion. For immunodepletion, 8 μg of anti-FAN1 antibody (Proteintech, cat # 17600-1-AP) or rabbit IgG control (ThermoFisher, cat # 10500C) was bound to 20 μL of protein Dynabeads Protein A (ThermoFisher, cat # 10001D) suspension and incubated with HCT116 nuclear extract (0.4 mg) in a total volume of 29 μL on ice for 1 h. Following incubation, the dynabeads were pulled down using a magnet and the supernatant was directly used for the repair reaction in the presence or absence of FAN1 (as described above, except that 100 μg of nuclear extract was used per reaction and the reactions were incubated at 37 °C for 40 min). The extent of FAN1 immunodepletion was evaluated by Western blot using anti-FAN1 antibodies (Proteintech, cat # 17600-1-AP).

Statistical analysis. Statistical significance was established by one way ANOVA analyses for multiple comparisons as described in the Figure Legends.

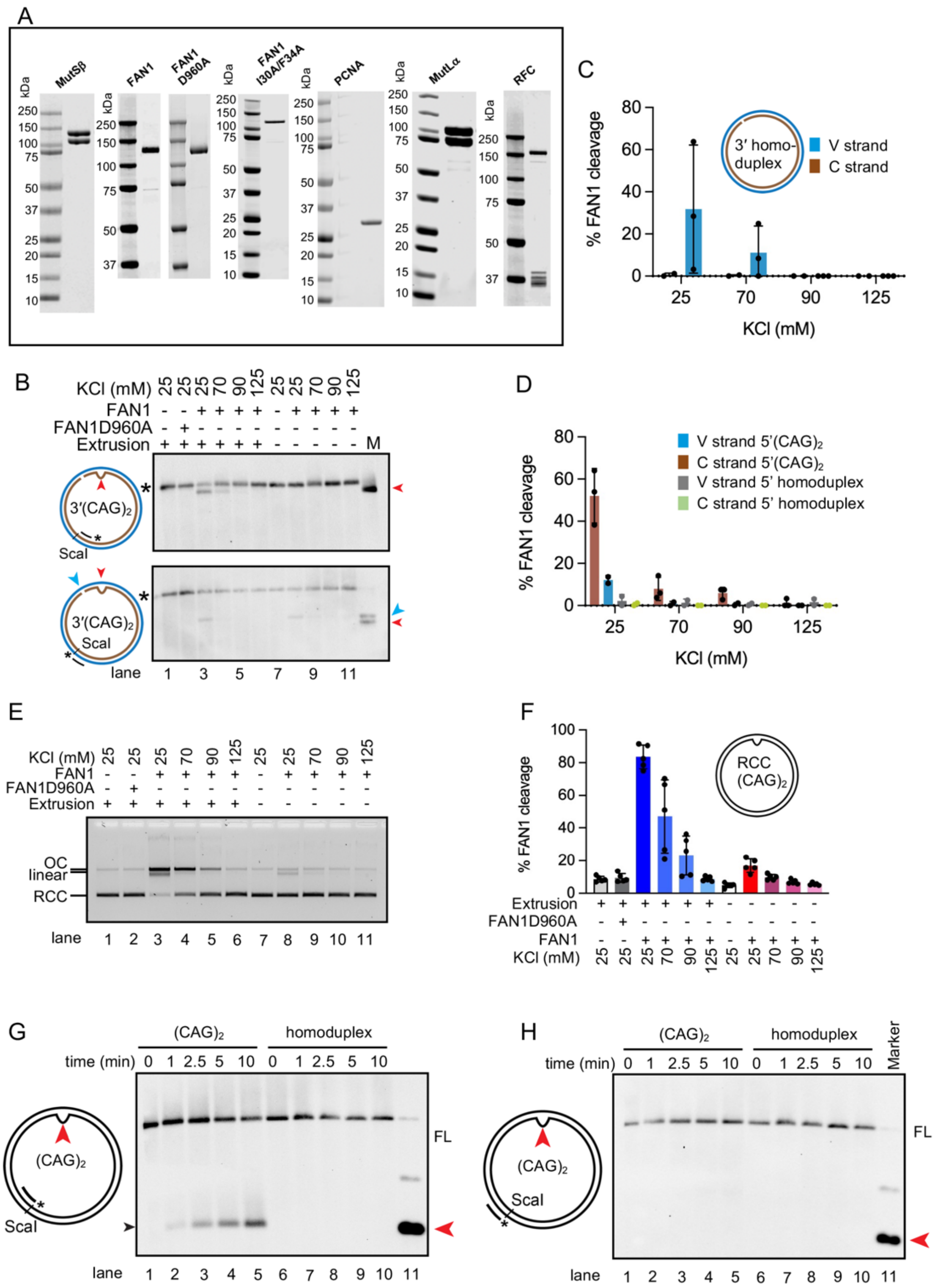

Fig. S1. PCNA and RFC activate FAN1 nuclease on DNA substrates harboring (CAG)$_2$ extrahelical extrusions. (**A**) Electrophoretic analysis of purified proteins used. (**B**) FAN1 cleavage on the 3′(CAG)$_2$ (lanes 1-6), or control homoduplex DNA substrate (lanes 7-11) at increasing ionic strength. Reaction products were linearized with ScaI, resolved on 1% alkaline agarose gels, followed by indirect end labeling with Fwd1947 probe (upper panel) or Rev1975

probe (lower panel). (**C**) Percentage of FAN1 cleavage on 3' homoduplex from experiments as in (**B**). Data are mean of three independent experiments ± SD. (**D**) Percentage of FAN1 cleavage at different ionic strengths on 5'$(CAG)_2$ or 5' homoduplex DNAs. Cleavage levels on the C strand of the 5'$(CAG)_2$ DNA are mean of 3 independent experiments ± SD, and on the V strand (and for 5' homoduplex) are average of 2 independent experiments with the error bars indicating ranges observed. (**E**) FAN1 activity on relaxed closed circular (RCC) $(CAG)_2$ extrusion or homoduplex control DNA substrates was evaluated at different KCl concentrations as indicated. The reaction products were resolved on 1% agarose gels in the presence of 1 µg/mL of EtBr. FAN1 cleavage is observed as the appearance of open circular (OC) or linear DNA. (**F**) Percentage of FAN1 cleavage on $(CAG)_2$ extrusion or homoduplex (as in **E**). Mean of 5 independent experiments with the error bars indicating SD. (**G**) 2.5 nM of RCC $(CAG)_2$ or homoduplex control DNAs were incubated with 22 nM FAN1 at 70 mM KCl, and the reactions were sampled as a function of time. DNA was digested with ScaI and resolved by electrophoresis on 1% alkaline agarose gels, followed by indirect end labeling with Fwd2020 probe to visualized the C-strand or (**H**) with Rev2047 probe to visualize the V-strand. Black arrow on the left indicates a preferential cleavage site. Red arrow on the right shows the approximate location of the $(CAG)_2$ extrusion.

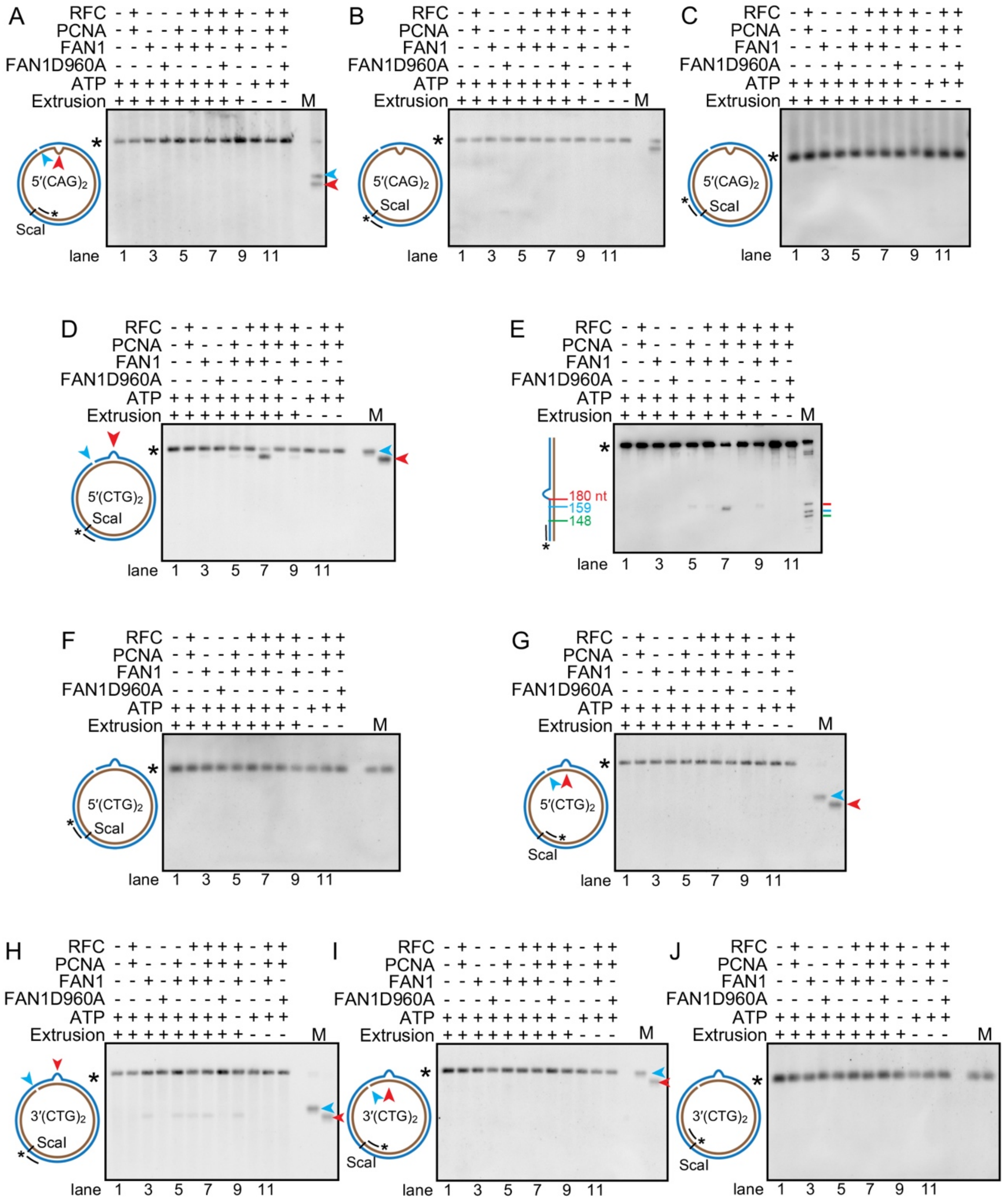

Fig. S2. Strand directionality of PCNA-, and RFC-dependent activation of the FAN1 nuclease. Representative images of strand break-, PCNA-, and RFC-activated FAN1 nuclease activity on 5'(CAG)$_2$ or 5' homoduplex control DNA substrates ((**A**)- Fwd1947 probe, (**B**)- Rev1975 probe, (**C**)- Rev2047 probe); 5'(CTG)$_2$ or 5' homoduplex DNAs ((**D**)-Rev1975 probe, (**E**)- Rev3056 probe, (**F**)- Rev2047 probe, (**G**)- Fwd1947 probe), or 3'(CTG)$_2$ or 3' homoduplex control ((**H**)- Rev1975 probe, (**I**)- Fwd1947 probe, (**J**)- Fwd2020 probe). M- marker- mr78 4xLacO plasmid was digested with BglII (to indicate the location of (CAG)$_2$ extrusion- red arrowhead) or BbvCI (to indicate the location of the nick- blue arrowhead). For (**E**) marker is as described in **Fig. 1F**. The mobility of the full length labeled DNA segment is indicated by asterisk.

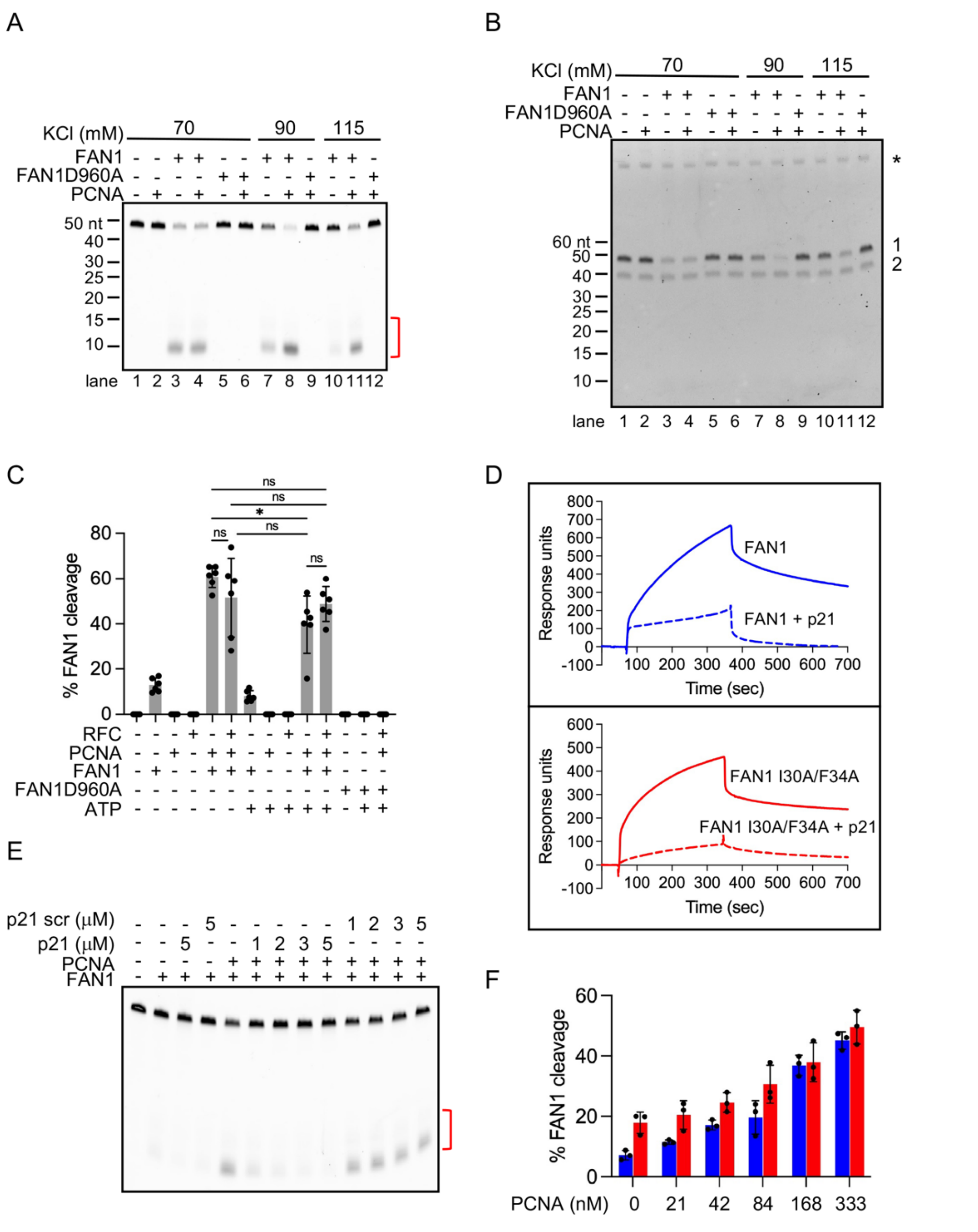

Fig. S3. FAN1 nuclease activity is promoted by PCNA on linear DNA substrates harboring extrahelical extrusions. (**A**) DNA substrate was incubated with FAN1 (or FAN1 D960A) at 70, 90, or 115 mM KCl in the presence or absence of PCNA as indicated, and the reaction products were resolved on 15% polyacrylamide gels containing 8 M urea and analyzed as described (SI Materials and Methods). Cleavage products are indicated by the red bracket. DNA size marker shown on the left; nt- nucleotides. (**B**) Gel from (**A**) was also stained with SYBR Safe to visualize both DNA strands. *partially denatured DNA, (1) DNA 3'-/Cy3/-labeled DNA strand containing (CAG)$_2$ extrusion, (2) complementary strand. The higher signal from 3'-/Cy3/-labeled DNA strand is likely due to binding of the SYBR Safe to /Cy3/. (**C**) FAN1 nuclease activity on linear DNA

requires PCNA but not RFC. FAN1 nuclease assays were performed on 3'-/Cy3/-labeled 40mer harboring $(CAG)_2$ extrusion (50 nM) at 115 mM KCl in the presence or absence of FAN1 (250 nM), RFC (60 nM), PCNA (333 nM as trimer), and 1 mM ATP as indicated. Products of the reactions were resolved on 15% polyacrylamide gels containing 8 M urea. Quantification based on 6 independent experiments. *$P<0.05$, 1-way ANOVA with post hoc Tukey's test. Error bars represent SD. (**D**) Assembly of FAN1-PCNA complex was scored by SPRS using CM5 sensor chip with immobilized PCNA (SI Materials and Methods). The top panel represents mass bound upon flow of 50 nM FAN1 (solid line) or a mixture of 50 nM FAN1 and 15 μM p21 peptide (dashed line). The bottom panel shows mass bound upon flow of 50 nM FAN1 I30A/F34A (solid line) or a mixture of 50 nM FAN1 I30A/F34A and 15 μM p21 peptide (dashed line). (**E**) p21 peptide inhibits PCNA activated FAN1. 3'-/Cy3/-labeled 40mer harboring $(CAG)_2$ extrusion was incubated with FAN1, PCNA, p21 peptide or p21 scrambled peptide (p21 scr), as indicated (SI Materials and Methods). Products of the reactions were resolved on 15% polyacrylamide gels containing 8 M urea. Cleavage products are indicated by the red bracket. (**F**) FAN1 (blue bars) or FAN1 I30A/F34A (red bars) nuclease activity on 3'-/Cy3/-labeled 40mer harboring $(CAG)_2$ extrusion DNAs was evaluated in the presence or absence of increasing concentrations of PCNA at 125 mM KCl (as described above). Quantification based on 3 independent experiments with error bars representing SD.

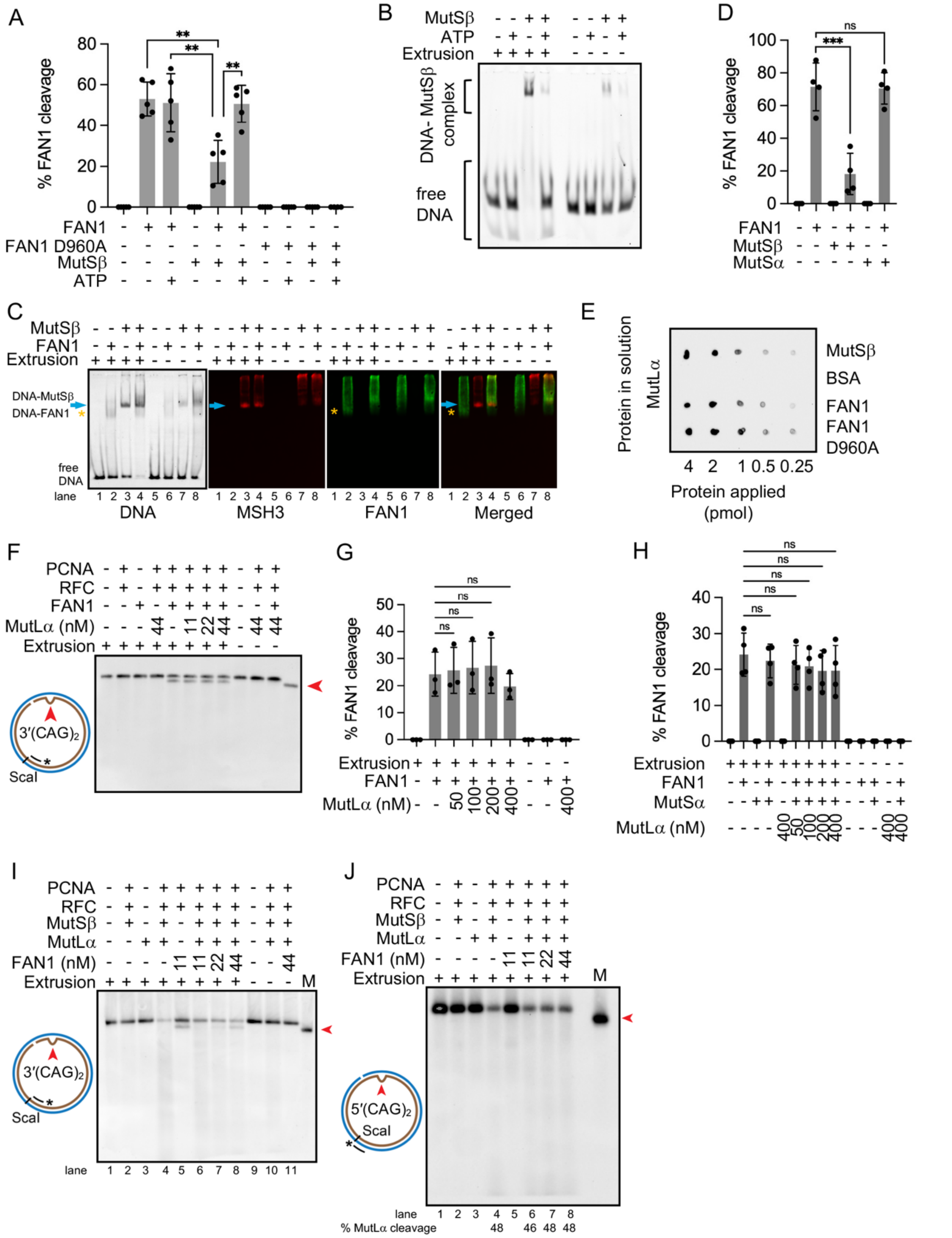

Fig. S4

Fig. S4. Effect of the components of DNA mismatch repair on FAN1 activity. (**A**) MutSβ modulates FAN1 activity by controlling the access to (CAG)2 extrusion. Quantification of percentage of FAN1 cleavage of 3'-/Cy3/-labeled (CAG)2 heteroduplex substrate in the presence or absence of MutSβ and ATP at 70 mM KCl (SI Materials and Methods). Five independent experiments were performed. **P<0.01, 1-way ANOVA with post hoc Tukey's test. Error bars represent SD. (**B**) 3'-/Cy3/-labeled (CAG)2 or homoduplex control were incubated with 125 nM

MutSβ (as indicated) in the presence or absence of ATP and resolved on 6% polyacrylamide gels. The position of free DNA and DNA-MutSβ complexes are indicated. (**C**) 3'-/Cy3/-labeled $(CAG)_2$ or homoduplex control were incubated with MutSβ and FAN1 (as indicated) and the reactions were resolved on 6% polyacrylamide gels. (**D**) 3'-/Cy3/-labeled $(CAG)_2$ DNA was preincubated with 125 nM of either MutSβ or MutSα on ice for 10 minutes. 250 nM of FAN1 was added and the reactions were incubated for an additional 5 minutes at 37 °C after which they were resolved on 15 % polyacrylamide gels containing 8 M urea and scored for FAN1 mediated cleavage (SI Materials and Methods). Graph is an average of four independent experiments with error bars indicating SD. ***$P<0.001$, 1-way ANOVA with post hoc Dunnett's test. (**E**) FAN1 interacts with MutLα. Indicated proteins were spotted onto nitrocellulose membrane and incubated with 0.36 μM MutLα overnight. MutLα was detected immunochemically (SI Materials and Methods). (**F**) $(CAG)_2$ extrusion containing DNA substrate was incubated with FAN1 or (FAN1 D960A), PCNA and RFC in the presence of increasing concentration of MutLα (as indicated). Indirect end labeling was performed with Fwd1947 probe. (**G**) Quantification of percentage of FAN1 activity on 3'-/Cy3/-labeled $(CAG)_2$ DNA substrate (or homoduplex control) in the presence of increasing concentration of MutLα at 70 mM KCl (SI Materials and Methods). Graph based on three independent experiments. ns- not significant, 1-way ANOVA with post hoc Dunnett's test (comparison to FAN1 in the absence of MutLα). Error bars represent SD. (**H**) Effect of MutSα and MutLα on FAN1 activity. 3'-/Cy3/-labeled $(CAG)_2$ substrate (or homoduplex control) were preincubated in the presence of 100 nM MutSα and/or MutLα (as indicated) for 10 minutes at 37 °C. Hundred nM FAN1 was added and the reactions were incubated for an additional 5 minutes after which they were terminated, resolved on 15 % polyacrylamide gels and scored for FAN1 activity as above (SI Materials and Methods). Data represents five independent experiments with error bars representing SD. ns- not significant. (**I**) The effect of FAN1 on MutLα endonuclease activity. Reaction samples from **Fig. 4D** were resolved on 1% alkaline agarose gels, followed by indirect end labeling using Fwd1947 probe. (**J**) 5'$(CAG)_2$ circular DNA substrate was incubated with PCNA, RFC, MutSβ, MutLα and increasing concentrations of FAN1 as indicated. Products of the reaction were digested with ScaI, resolved on 1% alkaline agarose gels, followed by indirect end labeling with Rev1975 probe. The experiment was repeated twice with a similar outcome.

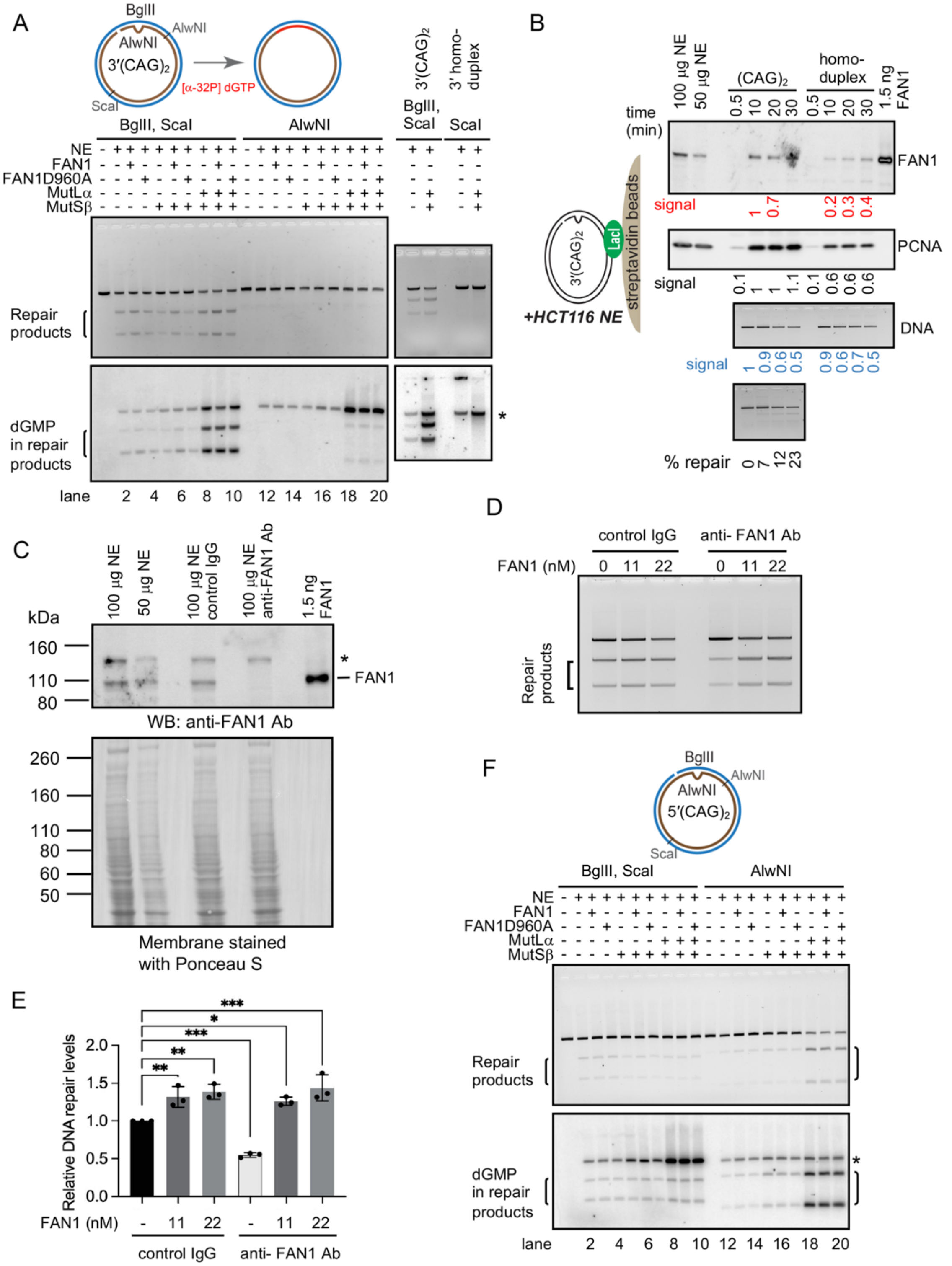

Fig. S5. DNA substrates containing (CAG)₂ extrahelical extrusion are subject to FAN1-dependent removal of extrahelical CAG extrusion in nuclear extracts. Heteroduplex DNA contains an extrahelical $(CAG)_2$ extrusion within overlapping recognition sites for BglII and AlwNI and a nick 181 nt 3' to the extrusion. DNA homoduplex control lacks the extrahelical extrusion. DNA substrates were incubated in the presence of [α-^{32}P] dGTP with nuclear extract derived from MLH1$^{-/-}$ MSH3$^{-/-}$ HCT116 cells, which was supplemented as indicated with FAN1, nuclease-

dead FAN1D960A, MutSβ, and MutLα (SI Materials and Methods). Repair was scored by cleavage with BglII and ScaI (bottom strand repair) or AlwNI (top strand repair), or with ScaI (for homoduplex) and electrophoresis on agarose gels. Repair products were visualized after staining with ethidium bromide (upper panel) while repair DNA synthesis was quantitated by exposure to phosphorimager screens (lower panel) (SI Material and Methods). Brackets shown on the left indicate repair products. Asterisk (*) indicates non-specific incorporation of dGMP into unrepaired DNA band, which also occurs in the homoduplex control sample. (**B**) Streptavidin beads immobilized 3'$(CAG)_2$ or 3' homoduplex DNA substrates (SI Materials and Methods) were incubated with HCT116 nuclear extracts (schematic on the left). Proteins bound to DNA substrates were analyzed by western blot. FAN1 was preferentially pulled down with 3'$(CAG)_2$ substrate compared to 3' homoduplex control (red numbers normalized to $(CAG)_2$ signal at 10 min). DNA recovery from each time point for both DNA substrates (numbers in blue normalized to DNA signal for $(CAG)_2$ at 0.5 min). Repair of $(CAG)_2$ substrate was evaluated by BglII, ScaI digestion at each time point with the percentage of repair shown below the image. Experiment was repeated 2 times with similar results. (**C**) Immunodepletion of FAN1 from HCT116 nuclear extracts. HCT116 nuclear extracts were incubated either with anti FAN1 antibody or control IgG (SI Materials and Methods). FAN1 levels were evaluated by Western blot with anti FAN1 antibody (upper panel). Recombinant FAN1 served as a positive control. Membrane was also stained with Ponceau S to visualize total protein levels in each lane (lower panel). (**D**) FAN1-immunodepleted (or mock-depleted) HCT116 nuclear extracts were incubated with 5'$(CAG)_2$ DNA substrate in the presence or absence of recombinant FAN1. Repair was scored after BglII, ScaI digestion. (**E**) Quantification is a mean of 3 independent experiments ± SD (data normalized to IgG only). *$P<0.05$, **$P<0.01$, ***$P<0.001$, 1-way ANOVA with post hoc Dunnett's test (comparison to IgG only). (**F**) Processing of heteroduplex DNA containing an extrahelical $(CAG)_2$ within overlapping recognition sites for BglII and AlwNI and a nick 178 nt 5' to the extrusion. Substrates were incubated in the presence of [α-^{32}P] dGTP with nuclear extract derived from MLH1$^{-/-}$ MSH3$^{-/-}$ HCT116 cells, which was supplemented as indicated with FAN1, nuclease-dead FAN1 D960A, MutSβ, and MutLα (SI Materials and Methods). Repair was scored by cleavage with BglII and ScaI (bottom strand repair) or AlwNI (top strand repair) and electrophoresis on agarose gels. Repair products were visualized after staining with ethidium bromide (upper panel) and repair DNA synthesis quantitated by exposure to phosphorimager screens (lower panel) (SI Materials and Methods). Brackets shown on the left indicate repair products. Asterisk (*) indicates non-specific incorporation of dGMP into unrepaired DNA band, which also occurs in the homoduplex control sample.

Table S1. Sequence inserts for plasmid constructs.

Plasmid name*	Sense strand sequence	Cloning site(s)
pGB1-mr77	5'TCGACCTAG**CAGCTGCTG**ATCTCGAGTCTAGAAATTCG	
pGB-mr78	5'TCGACCTAGC**AGATCT**CGAGTCTAGAAATTCG	
pGB1-mr77 4xLacO	5'GCT***AATTGTGAGCGGATAACAATT***GTTAGGGAGG***AATTGTGAGCGGATAACAATT***TGGAG TTGAT***AATTGTGAGCGGATAACAATT***GGCTTCAACGT***AATTGTGAGCGGATAACAATT***GCTCTTCC	SapI
pGB1-mr78 4xLacO	5'GCT***AATTGTGAGCGGATAACAATT***GTTAGGGAGG***AATTGTGAGCGGATAACAATT***TGGAG TTGAT***AATTGTGAGCGGATAACAATT***GGCTTCAACGT***AATTGTGAGCGGATAACAATT***GCTCTTCC	SapI

Entries 1 and 2 are phagemid pGEM-7Zf(-) (Promega) derivatives as described previously (9). Bold characters indicate restriction sites used to score heteroduplex repair. Entries 3 and 4: Four tandem copies of Lac operator sequences (LacO) (bold italic) (12) were cloned into SapI restriction site of pGB-mr77 or pGB1-mr78 resulting in pGB1-mr77 4xLacO or pGB1-mr78 4xLacO, respectively. Additional G to A mutation was introduced during cloning (position 376 or 370 for pGB1-mr77 4xLacO or pGB1-mr78 4xLacO, respectively).

Table S2. DNAs used for substrate construction.

Extrahelical element or homoduplex	ssDNA (sense strand)	RF DNA
$(CTG)_2$	pGB1-mr77 4xLacO	pGB1-mr78 4xLacO
$(CAG)_2$	pGB1-mr78 4xLacO	pGB1-mr77 4xLacO
Homoduplex	pGB1-mr78 4xLacO	pGB1-mr78 4xLacO

Table S3. DNA oligonucleotides used for substrate preparation and indirect end labeling

Oligo	Sequence	Description
top-mr78	5'CTCTAGACTCGACCTAGCAGATCTCGAGTCTAGAAATTCG	
top-$(CTG)_2$-mr78	5'CTCTAGACTCGACCTAGCAG$(CTG)_2$ATCTCGAGTCTAGAAATTCG	3'-/Cy3/-labeled homoduplex was obtained by annealing top-$(CTG)_2$-mr78 and bottom-$(CAG)_2$-mr78
bottom-$(CAG)_{2,13}$-mr78	5'CGAATTTCTAGACTCGAGAT$\underline{(CAG)}_{2,13}$CTGCTAGGTCGAGTCTAGAG/Cy3/	3'-/Cy3/-labeled $(CAG)_{2,13}$ substrate was obtained by annealing of bottom-$(CAG)_{2,13}$-mr78 and top-mr78
bottom-$(CTG)_{2,13}$-mr78	5'CGAATTTCTAGACTCGAGAT$\underline{(CTG)}_{2,13}$CTGCTAGGTCGAGTCTAGAG/Cy3/	3'-/Cy3/-labeled $(CTG)_{2,13}$ substrate was obtained by annealing of bottom-$(CTG)_{2,13}$-mr78 and top-mr78
bottom-AGCCTA-mr78	5'CGAATTTCTAGACTCGAGAT$\underline{\text{AGCCTA}}$CTGCTAGGTCGAGTCTAGAG/Cy3/	3'/Cy3/ labeled AGCCTA substrate was obtained by annealing of bottom-AGCCTA-mr78 and top-mr78
/5DigN/mr78Fwd2020	/5DigN/GTGTATGCGGCGACCGAGTTGCTCTTG	Target strand: C strand (brown)
/5DigN/mr78Rev2047	/5DigN/CAAGAGCAATCGGTCGCCGCATACAC	Target strand: V strand (blue)
/5DigN/mr78Fwd1947	/5DigN/CATAATTCTCTTACTGTCATGCCATCCG	Target strand: C strand (brown)
/5DigN/mr78Rev1975	/5DigN/CGGATGGCATGACAGTAAGAGAATTATG	Target strand: V strand (blue)
/5DigN/mr78Fwd3028	/5DigN/GGGGATGTGCTGCAAGGCGATTAAGTTG	Target strand: C strand (brown)
/5DigN/mr78Rev3056	/5DigN/CAACTTAATCGCCTTGCAGCACATCCCC	Target strand: V strand (blue)

Entries 3, 4, 5: The underlined sequence indicates the sequence of the DNA extrahelical extrusion after annealing DNA complementary strands, as indicated.

SI References

1. L. J. Blackwell, S. Wang, P. Modrich, DNA chain length dependence of formation and dynamics of hMutSalpha.hMutLalpha.heteroduplex complexes. *The Journal of biological chemistry* **276**, 33233-33240 (2001).
2. J. Genschel, S. J. Littman, J. T. Drummond, P. Modrich, Isolation of MutSbeta from human cells and comparison of the mismatch repair specificities of MutSbeta and MutSalpha. *The Journal of biological chemistry* **273**, 19895-19901 (1998).
3. Y. Matsumoto, R. C. Brooks, A. Sverzhinsky, J. M. Pascal, A. E. Tomkinson, Dynamic DNA-bound PCNA complexes co-ordinate Okazaki fragment synthesis, processing and ligation. *J Mol Biol* **432**, 166698 (2020).
4. J. M. Gulbis, Z. Kelman, J. Hurwitz, M. O'Donnell, J. Kuriyan, Structure of the C-terminal region of p21(WAF1/CIP1) complexed with human PCNA. *Cell* **87**, 297-306 (1996).
5. K. Kratz *et al.*, Deficiency of FANCD2-associated nuclease KIAA1018/FAN1 sensitizes cells to interstrand crosslinking agents. *Cell* **142**, 77-88 (2010).
6. T. Liu, G. Ghosal, J. Yuan, J. Chen, J. Huang, FAN1 acts with FANCI-FANCD2 to promote DNA interstrand cross-link repair. *Science* **329**, 693-696 (2010).
7. R. Parsons *et al.*, Hypermutability and mismatch repair deficiency in RER+ tumor cells. *Cell* **75**, 1227-1236 (1993).
8. M. L. Phipps *et al.*, Beyond Helper Phage: Using "Helper Cells" to Select Peptide Affinity Ligands. *PloS one* **11**, e0160940 (2016).
9. A. Pluciennik *et al.*, Extrahelical (CAG)/(CTG) triplet repeat elements support proliferating cell nuclear antigen loading and MutLalpha endonuclease activation. *Proceedings of the National Academy of Sciences of the United States of America* **110**, 12277-12282 (2013).
10. E. Warbrick, D. P. Lane, D. M. Glover, L. S. Cox, A small peptide inhibitor of DNA replication defines the site of interaction between the cyclin-dependent kinase inhibitor p21WAF1 and proliferating cell nuclear antigen. *Current biology : CB* **5**, 275-282 (1995).
11. J. Zhang *et al.*, DNA interstrand cross-link repair requires replication-fork convergence. *Nature structural & molecular biology* **22**, 242-247 (2015).
12. J. M. Dewar, M. Budzowska, J. C. Walter, The mechanism of DNA replication termination in vertebrates. *Nature* **525**, 345-350 (2015).
